# Economic Evaluation of Oral Nirmatrelvir-Ritonavir for COVID-19 in Higher Risk Outpatients

**DOI:** 10.1001/jamanetworkopen.2026.12381

**Published:** 2026-05-06

**Authors:** May Ee Png, Victoria Harris, Ly-Mee Yu, Paul Little, F. D. Richard Hobbs, Christopher C. Butler, Stavros Petrou

**Affiliations:** 1 Nuffield Department of Primary Care Health Sciences, University of Oxford, Oxford, United Kingdom; 2 Nuffield Department of Orthopaedics, Rheumatology and Musculoskeletal Sciences, University of Oxford, Oxford, United Kingdom; 3 Primary Care Research Centre, University of Southampton, Southampton, United Kingdom; 4 Oxford Institute of Digital Health, University of Oxford, Oxford, United Kingdom

## Abstract

**Importance:**

Nirmatrelvir-ritonavir is an oral antiviral recommended for treating high-risk individuals with COVID-19 in the community. However, there remains uncertainty over its cost-effectiveness in largely vaccinated populations and subgroups defined by age and clinical risk.

**Objective:**

To compare the cost-effectiveness of nirmatrelvir-ritonavir vs usual care based on the UK Platform Adaptative Trial of Novel Antivirals for Early Treatment of COVID-19 in the Community (PANORAMIC) trial.

**Design, Setting, and Participants:**

This economic evaluation was a within-trial cost-utility analysis conducted using data from the PANORAMIC trial and linked routine health care data from December 8, 2021, to September 30, 2024, adopting a UK National Health Service (NHS) and personal social services perspective over 6 months. Eligible participants were community-dwelling adults aged 50 years and older or ages 18 to 49 years with comorbidities. Data were analyzed from April 20, 2022, to September 30, 2024, for the nirmatrelvir-ritonavir group and December 8, 2021, to September 30, 2026, for usual care.

**Intervention:**

Oral nirmatrelvir-ritonavir.

**Main Outcome and Measures:**

Cost, quality-adjusted life-years (QALYs), and incremental cost-effectiveness ratio (ICER) of nirmatrelvir-ritonavir vs usual care.

**Results:**

There were 1736 participants randomized to receive nirmatrelvir-ritonavir plus usual care (mean [SE] age, 54.7 [0.29] years), and 1768 to usual care alone (mean [SE] age, 54.8 [0.28] years); 2405 in the total cohort were female (69.2%]), and 3455 (98.6%) were vaccinated. Nirmatrelvir-ritonavir was cost-effective, on average, with lower total costs (£124; 95% CI, –£1207 to £1455) and higher QALYs (0.0110; 95% CI, 0.0062-0.0170) over 6 months compared with usual care (ICER, £10 897 per QALY). Nirmatrelvir-ritonavir had a 0.65 probability of being cost-effective at a £20 000 per QALY cost-effectiveness threshold, indicating decision uncertainty. The intervention remained cost-effective in most sensitivity analyses and prespecified subgroups, although several subgroup analyses were based on small sample sizes and should be interpreted as exploratory.

**Conclusions and Relevance:**

In this economic evaluation of nirmatrelvir-ritonavir for COVID-19, cost-effectiveness varied by age, comorbidity, and vaccination status. Subgroup findings should be interpreted with caution, but targeted prescribing for at-risk adults aged 18 to 64 years, those aged 65 to 69 years, and adults aged 75 years and above, particularly those with comorbidities and not recently vaccinated, was likely to provide the greatest health system value; cost-effectiveness was not observed in low-risk adults aged 50 to 64 years, those aged 70 to 74 years, or in unvaccinated or recently vaccinated individuals.

## Key Points

**Question:**

Is treatment with oral nirmatrelvir-ritonavir cost-effective compared with usual care for higher risk outpatients with COVID-19 in the UK?

**Findings:**

In this economic evaluation, the value for money of oral nirmatrelvir-ritonavir differed across population risk strata, with favorable economic outcomes concentrated among individuals at higher clinical risk in a highly vaccinated setting.

**Meaning:**

These results suggest that routine prescribing of nirmatrelvir-ritonavir is unlikely to be cost-effective in older adults, but targeted use in clearly defined high-risk groups may represent a more efficient strategy for the health system.

## Teaser

This economic evaluation examines the cost-effectiveness of nirmatrelvir-ritonavir vs usual care for COVID-19 treatment in a UK clinical trial cohort of high-risk adults.

## Introduction

Nirmatrelvir-ritonavir has been approved and is widely recommended for early treatment in high-risk COVID-19 patients,^1^ supported by evidence from clinical trials and population studies demonstrating reduced risk of hospitalization and death.^2,3,4^ In the EPIC-HR trial, nirmatrelvir-ritonavir reduced the risk of COVID-19 hospitalization or death by 89% when initiated within 5 days of symptom onset.^2^ In response to accumulating evidence, the World Health Organization issued a recommendation on April 22, 2022, for its use in COVID-19 patients with severe underlying conditions.^5^

Numerous decision analytic model-based economic evaluations^6,7,8,9,10,11,12,13,14,15,16,17,18^ and cohort-based economic evaluations^19,20^ across high-income and middle-income countries consistently showed that nirmatrelvir-ritonavir was cost-effective or cost-saving during the Omicron wave when targeted at high-risk outpatients, particularly older adults and individuals with comorbidities. However, findings on its cost-effectiveness by vaccination status have been more variable. For example, Nilsson et al^16^ concluded that nirmatrelvir-ritonavir was cost-effective regardless of the vaccination status of the recipient. Wikman-Jorgensen et al^12^ reported that it was cost-effective primarily when targeted at unvaccinated, high-risk individuals, while Savinkina et al^6^ found that treatment among vaccinated individuals can be cost-effective, depending on assumptions about efficacy, cost, and hospitalization risk.

However, all prior economic evaluations were nonrandomized and prone to confounding, in addition to being conducted in settings with different health care financing arrangements and population characteristics, which limits the generalizability of their findings. To address this, we conducted a trial-based economic evaluation, leveraging the high internal validity of a randomized clinical trial to estimate the short and medium-term costs and outcomes of nirmatrelvir-ritonavir vs usual care in higher-risk, nonhospitalized adult patients with COVID-19 in the UK setting.

## Methods

### Study Design and Population

This economic evaluation was conducted alongside the Platform Adaptative Trial of Novel Antivirals for Early Treatment of COVID-19 in the Community (PANORAMIC) trial (ISRCTN30448031), an open-label, adaptive platform trial evaluating the effectiveness of novel community-based treatments for COVID-19 across the UK. The base case economic evaluation was conducted over 6 months under the UK National Health Service (NHS) and Personal Social Services (PSS) perspective, consistent with National Institute for Health and Care Excellence (NICE) reference case requirements.^21^ Comprehensive trial details, including sample size calculations, recruitment methods and clinical outcomes, have been published previously.^22^ The trial population included adults aged 50 years or older regardless of comorbidity status, or adults aged 18 to 49 years with specified comorbidities associated with increased risk of severe COVID-19. Participants were randomly assigned via a secure web-based system using equal allocation, stratified by age (younger than 50 years vs 50 years and older) and COVID-19 vaccination status (at least 1 dose 14 days or more before randomization vs otherwise), to receive oral nirmatrelvir 300 mg (in two 150 mg tablets) with ritonavir 100 mg, taken together twice daily for 5 days, in addition to usual care, or usual care only.

This economic evaluation compared nirmatrelvir-ritonavir with usual care over the periods (April 20, 2022, to September 30, 2024, for nirmatrelvir-ritonavir; December 8, 2021, to September 30, 2024, for usual care). The health economic methods used here build on previous work conducted within the same platform trial.^23^ This economic evaluation follows the Consolidated Health Economic Evaluation Reporting Standards (CHEERS) reporting guideline to ensure transparency and relevance for decision-makers and stakeholders. The economic evaluation was conducted under the ethical approvals obtained for the main trial from South Central-Berkshire Research Ethics Committee and was prespecified in a health economic analysis plan (HEAP), approved by the trial steering committee prior to unmasking. All participants provided written informed consent for participation in the trial, including use of their data for the economic evaluation. All analyses were performed in R version 4.5 (R Project for Statistical Computing).^24^

### Resource Use and Costing

Health care and PSS resource use data were obtained from participant self-reported 28-day daily diaries, 3-month and 6-month questionnaires, and for hospital service resource use linked routinely collected data, covering a retrospective period dating back to 2021 from all 4 UK nations: Hospital Episode Statistics (HES) for England (October 2024 dataset), the Secure Anonymised Information Linkage (SAIL) Databank for Wales (September 2024), the electronic Data Research and Innovation Service (eDRIS) for Scotland (September 2024), and the Honest Broker Service (HBS) in Northern Ireland (October 2023). Categories of resource use included primary and secondary health care services, personal social services and prescription medications. Participants also reported time off work due to symptoms associated with COVID-19 (ie, productivity loss) in the 3-month and 6-month follow-up questionnaires, which were analyzed separately from the NHS and PSS base case.

Discrepancies in resource use estimates were resolved using a prespecified hierarchy, described in detail in Png et al.^23^ All resource use was valued in 2023-24 GBP using nationally recognized UK unit costs relevant at the time of analysis (eTable 1 in Supplement 1). Where applicable, historical costs were inflated to 2023-24 prices using the Personal Social Services Research Unit (PSSRU) Hospital and Community Health Services (HCHS) Index.^25^ Hospital-based resource use, including inpatient admissions (elective and nonelective), day cases, emergency department attendances, and outpatient visits, was costed using Healthcare Resource Group (HRG) codes mapped to the 2023-24 National Cost Collection.^26^ Community-based health and social care services were valued using PSSRU Unit Costs of Health and Social Care,^25^ and medication prices were taken from the Prescription Cost Analysis database.^27^ The unit cost of nirmatrelvir-ritonavir was obtained from the British National Formulary.^28^ Costs associated with routine prescribing and dispensing activities, including pharmacist time, were assumed to be captured within national unit costs for prescription medications and were not costed separately, consistent with an NHS and PSS perspective. Productivity losses were included for participants in paid employment only and were valued using a human capital approach, applying the UK median national wage reported by the Office for National Statistics.^29,30^ No discounting was applied to costs and outcomes due to the short within-trial time horizon.

### Outcomes Measurement and Valuation

Health-related quality of life was measured using the EQ-5D-5L (EuroQol 5-Dimension 5-Level) instrument at baseline, 14-day, 28-day, and 3-month and 6-month follow-up.^31^ Utility scores were derived from responses to the EQ-5D-5L descriptive system. In line with current recommendations by NICE,^21^ and in the absence of a NICE-recommended UK EQ-5D-5L value set for reference case analyses, EQ-5D-5L responses were converted to utility values using a validated mapping function onto the UK EQ-5D-3L value set, developed by the NICE Decision Support Unit.^32^

Quality-adjusted life years (QALYs) were estimated using the area-under-the-curve approach, combining utility values and observed participant survival status over the trial follow-up period. For the base case analysis, QALYs were calculated using the trapezoidal rule,^33^ with adjustment for baseline utility.

### Handling of Missing Data

Missing cost and utility data were addressed using multiple imputation by chained equations, implemented via seemingly unrelated regression (SUR), following the methodology proposed by Ben et al.^34^ This approach allows for the simultaneous imputation of correlated outcomes (eg, costs and QALYs), improving efficiency and preserving the joint distribution of economic data. The imputation model included baseline covariates, observed EQ-5D-5L utilities and scores from the EQ-5D-5L visual analogue scale, as well as health care resource use variables.

Ten imputed datasets were generated and analyzed separately, with estimates pooled using Rubin’s rules to account for within- and between-imputation variance.^35^ The imputation was conducted under the missing-at-random assumption.

### Patient and Public Involvement

The PANORAMIC trial incorporated patient and public involvement (PPI) throughout, including in refining the research question and in the design and delivery of participant-facing materials, as detailed in the trial protocol.^39^ We also plan to share the main findings with trial participants and the wider public, and will engage the PANORAMIC trial’s PPI representatives to support the interpretation of results and the development of appropriate dissemination strategies.

### Statistical Analysis

#### Main Analysis

Resource use and costs were summarized by treatment group and time point of patient assessment. Differences in continuous outcomes were assessed using *t* tests, and categorical outcomes via Pearson χ^2^ tests. Means and standard errors for cost components and QALYs were calculated, with uncertainty estimated using nonparametric bootstrapping (1000 replications). A 2-sided significance level of .05 was used throughout. Incremental cost-effectiveness ratios (ICERs) and net monetary benefit (NMB) statistics were calculated from the NHS and PSS perspective. SUR models were applied to jointly estimate total costs and QALYs, accounting for their correlation. Models were adjusted for randomization stratification factors (age, vaccination status, and comorbidity status).

Cost-effectiveness was assessed in accordance with the intention-to-treat (ITT) principle using cost-effectiveness thresholds of £15 000, £20 000, and £30 000 per QALY gained, consistent with NICE guidance and recent UK health care decision-making trends.^36,37^ Decision uncertainty was characterized using cost-effectiveness acceptability curves (CEACs) derived from bootstrap replications.

#### Sensitivity and Subgroup Analyses

Two sensitivity analyses were conducted: adopting a societal perspective by including values for productivity losses; and a complete case analysis to assess the impact of missing data. These analyses used the same imputation and SUR framework as the base case.

Prespecified subgroup analyses were conducted to explore heterogeneity in the cost-effectiveness of nirmatrelvir-ritonavir across patient characteristics, defined a priori in the Master Statistical Analysis Plan (MSAP) and HEAP,^22^ and have been described in detail in a previously published economic evaluation framework.^38^ Each subgroup analysis compared nirmatrelvir-ritonavir with usual care within the defined subgroup and was conducted to assess treatment effect heterogeneity rather than to represent mutually exclusive implementation strategies. Accordingly, subgroup results are presented as within-subgroup incremental cost-effectiveness estimates and were not ranked or evaluated using a sequential incremental approach across subgroups.

Subgroups were defined by age (using 65 and 80 years as cutoffs), presence of comorbidities (including lung disease, obesity, diabetes, cardiovascular disease, and immune disorders), vaccination status (including number of doses and time since last dose), symptom duration and severity at baseline, swab test modality (polymerase chain reaction vs lateral flow device), use of inhaled corticosteroids, and risk category (from 2 to 9) where those in categories 6 to 9 consisted of an at-risk group. An at-risk group was defined as having 1 or more of the underlying chronic health conditions considered to make them clinically at-risk as listed in the inclusion criteria for the trial.

## Results

### Study Participants

There were 1736 participants randomized to receive nirmatrelvir-ritonavir plus usual care, and 1768 to usual care alone. The mean (SE) age of participants was 54.7 (0.29) years in the nirmatrelvir-ritonavir group and 54.8 (0.28) years in the usual care group (eTable 2 in Supplement 1). The distribution of baseline characteristics was broadly similar between groups. The majority of participants were female (2405 of 3504 [68.6%]), had 1 or more comorbid conditions (2312 of 3504 [66.0%]), and were vaccinated (3455 of 3504 [98.6%]).

### Completeness of Resource Use and EQ-5D-5L Data

Based on completion rates for resource use (from an NHS and PSS perspective) and EQ-5D-5L responses from baseline to 6 months postrandomization, 1273 participants (73.3%) in the nirmatrelvir-ritonavir group and 1213 (68.6%) in the usual care arm had complete data across all time points (eTable 3 in Supplement 1). The pattern of missing data was nonmonotonic, with some participants missing data at intermediate follow-ups but completing later assessments.

### Health and Social Care Resource Use and Time Off Work

In the available case analysis, there were no statistically significant differences in overall health and social care resource use between the nirmatrelvir-ritonavir and usual care groups across the full follow-up period (eTable 4 in Supplement 1). However, several differences were observed during specific time windows.

Within the first 28 days postrandomization, participants in the nirmatrelvir-ritonavir group reported fewer NHS 111 contacts (mean difference, −0.0420; 95% CI, −0.0669 to −0.0177; *P* < .001); hospital at-home contacts (−0.0647; 95% CI, −0.1329 to −0.0100; *P* = .04); and primary care prescriptions (−0.0807; 95% CI, −0.1180 to −0.0428; *P* < .001) compared with the usual care group. They also reported fewer free-text–recorded contacts with other services (−0.0305; 95% CI, −0.0533 to −0.0094; *P* = .008).

Between 28 days and 3 months, those in the nirmatrelvir-ritonavir group had fewer primary care prescriptions (−0.1211; 95% CI, −0.2211 to −0.0206; *P* = .02) compared with the usual care group. Likewise, between 3 and 6 months, those in the nirmatrelvir-ritonavir group had fewer primary care prescriptions (−0.0762; 95% CI, −0.1440 to −0.0077; *P* = .03) compared with the usual care group.

### Health and Personal Social Service Costs

Across all follow-up periods, admitted patient care and critical care costs were the largest contributors to total costs in both treatment groups (eTable 5 in Supplement 1). Over the 6-month follow-up, total costs were similar between groups, with no significant difference in overall health care costs.

During the first 28 days, small absolute differences in specific cost components were observed, with slightly lower mean costs in the nirmatrelvir-ritonavir group for NHS 111 services, hospital at-home services, primary care prescriptions, and other service contacts. However, these differences were modest in magnitude (£5 or less per category) and did not meaningfully affect total costs. Between 28 days and 3 months, as well as between 3 and 6 months, there were no statistically significant differences in mean cost of health and personal social services between the nirmatrelvir-ritonavir and usual care groups.

### Health Utilities

In the available case analysis of EQ-5D-5L utility, participants in the nirmatrelvir-ritonavir group reported significantly higher mean health utility scores than those in the usual care group at 14 days (mean difference, 0.0241; 95% CI, 0.0103-0.0380; *P* < .001) and 28 days (0.0153; 95% CI: 0.0014-0.0294; *P* = .03) (eTable 6 in Supplement 1). No statistically significant differences in EQ-5D-5L utility scores were observed at other time points. In addition, EQ-5D-5L visual analogue scale (VAS) scores were consistently higher in the nirmatrelvir-ritonavir group compared with usual care at all time points, with statistically significant differences observed at 14 days (*P* = .005).

### Cost-Effectiveness Results

From the base case, the mean (SE) total cost per participant was £3253 (£753) in the nirmatrelvir-ritonavir group and £3129 (£439) in the usual care group, resulting in an incremental cost of £124 (95% CI, −£1207 to £1455) (Table). The mean (SE) QALYs per participant were 0.4146 (0.0018) in the nirmatrelvir-ritonavir group and 0.4032 (0.0015) in the usual care group, yielding an incremental QALY gain of 0.0110 (95% CI, 0.0062-0.0170).

**Table.** Base Case Cost-Effectiveness of Nirmatrelvir-Ritonavir vs Usual Care Over 6 Months, in 2023 to 2024 Prices^a^

Measure	Mean (SE)		Difference, mean (95% CI)
	Nirmatrelvir-ritonavir (n = 1736)	Usual care (n = 1768)	
Costs, £	3253 (753.4)	3129 (439.0)	124 (−1207 to 1455)
QALYs	0.4146 (0.0018)	0.4032 (0.0015)	0.0110 (0.0062-0.0170)
ICER, £/QALY	NA	NA	10 897 (NE quad)
Probability of nirmatrelvir-ritonavir being cost-effective at			
£15 000/QALY	NA	NA	0.61
£20 000/QALY	NA	NA	0.65
£30 000/QALY	NA	NA	0.72

Abbreviations: ICER, incremental cost-effectiveness ratio; NA, not applicable; QALY, quality-adjusted life-year.

^a^ Incremental estimates and 95% CIs were derived using nonparametric bootstrapping.

The resulting ICER was £10 897 per QALY gained, with most bootstrap replicates below the £30 000 per QALY threshold in the cost-effectiveness plane (Figure, A). The NMB plot (Figure, B) indicated a high probability of cost-effectiveness for nirmatrelvir-ritonavir across the studied cost-effectiveness thresholds. At a cost-effectiveness threshold of £20 000 per QALY, the probability that nirmatrelvir-ritonavir is cost-effective was 0.65, increasing to 0.72 at a cost-effectiveness £30 000 threshold (Figure, C).

**Figure.**
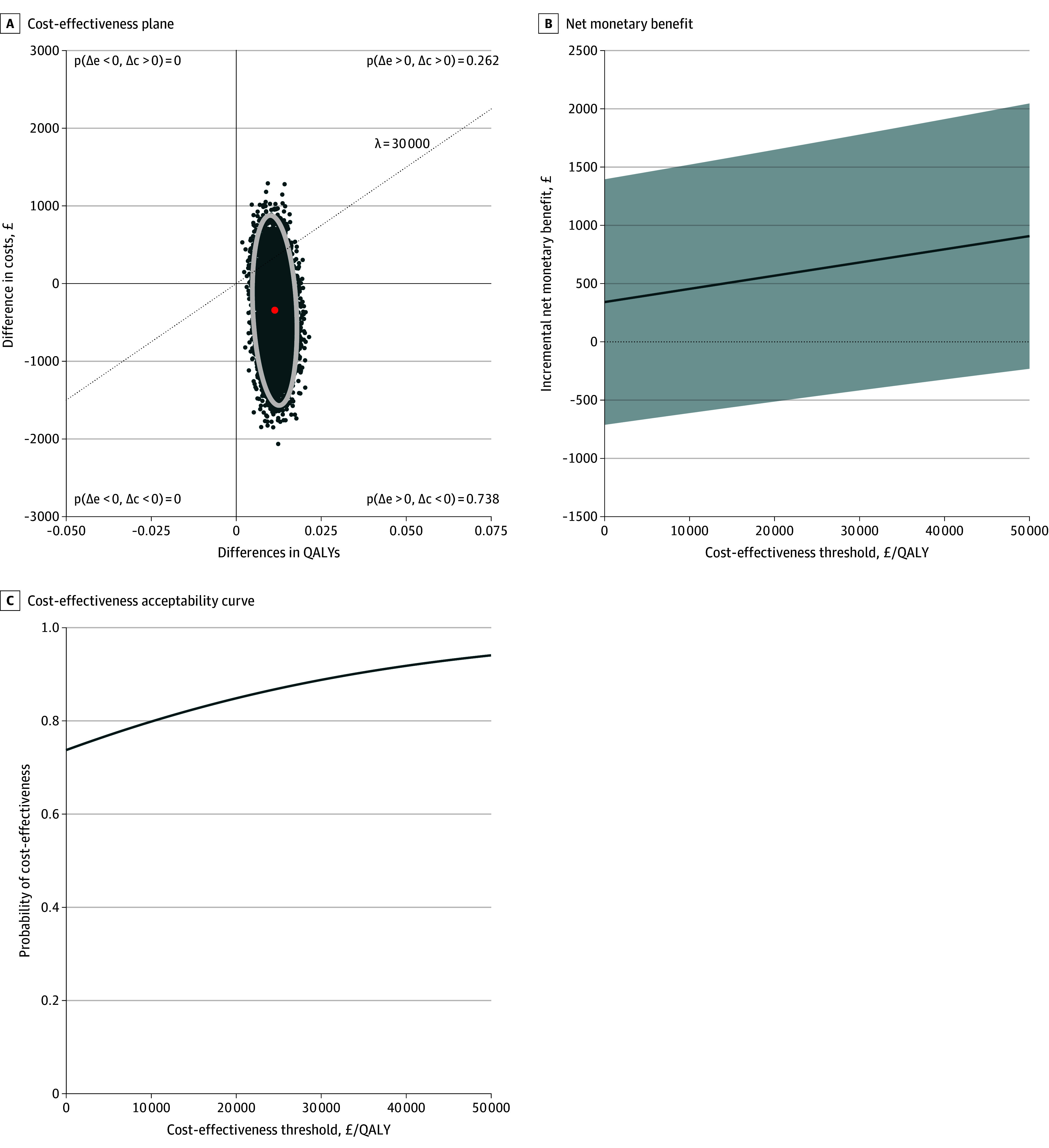
Cost-Effectiveness of Nirmatrelvir-Ritonavir vs Usual Care in the PANORAMIC Trial A, Dotted line indicates the £30 000 per QALY cost-effectiveness threshold; red dot, the mean incremental cost and mean incremental QALY estimate; white ellipse, the joint uncertainty (95% uncertainty region) around the mean incremental cost-QALY estimate based on simulated cost-effectiveness pairs. B, Shaded area indicates 95% CI around the incremental net monetary benefit.

At a £20 000 per QALY threshold, the probability that nirmatrelvir-ritonavir is cost-effective was similar to the base case under a societal perspective (0.68 vs 0.65) but much lower in the complete-case analysis (0.14). Prespecified subgroup analyses were exploratory and showed heterogeneity in cost-effectiveness across population groups. The probability of nirmatrelvir-ritonavir being cost-effective was high in participants with comorbidities (0.86), lung disease (0.94), immune disorders (0.90), and among those prescribed inhaled corticosteroids (0.92), and in NHS risk categories 2, 3, 5, and 6 (age 80 years or older, 0.59; 75 to 79 years, 0.59; 65 to 69 years, 0.87; and 18 to 64 years at-risk, 0.87). In contrast, probabilities were low for those without comorbidities (0.07), without lung disease (0.27), not prescribed inhaled corticosteroids (0.26), and for NHS risk categories 4 and 7 through 9 (age 70 to 74 years, 0.01; 60 to 64 years not at-risk, 0.29; 55 to 59 years not at-risk, 0.29; 50 to 54 years not at-risk, 0.07).

Subgroup estimates based on smaller sample sizes (fewer than 100 participants per group), including adults aged 80 years or older, unvaccinated or partially vaccinated participants, and the smallest NHS risk categories, were imprecise and should be interpreted with caution. Vaccination patterns were mixed; cost-effectiveness was low in the unvaccinated (0.21), in people vaccinated within 3 months (0.27), and with 4 doses (0.23), but high with 1 to 3 doses (0.85, 0.77, 0.76) and when the most recent dose was 3 to 6 months (0.78) or more than 6 months prior (0.61). Results by simple age cut-off at 80 years were favorable in both strata (younger than 80 years, 0.63; 80 years or older, 0.67). Within older adults, probabilities varied by risk category, with low probabilities confined to ages 70 to 74 years. Detailed subgroup estimates are presented in the eTable 7 in Supplement 1, as these analyses are exploratory and not intended to support definitive subgroup-specific inference.

## Discussion

This economic evaluation from a large, prospective randomized clinical trial found that nirmatrelvir-ritonavir was cost-effective compared with usual care over a 6-month time horizon. Findings were robust in the societal perspective but attenuated in the complete-case analysis. Subgroup analyses revealed substantial heterogeneity: cost-effectiveness was consistently observed among individuals with comorbidities, lung disease, immune disorders, or those prescribed inhaled corticosteroids, and in NHS risk categories 2, 3, 5, and 6 (ages 80 years or older, 75 to 79 years, 65 to 69 years, and 18 to 64 years in an at-risk group). In contrast, probabilities of cost-effectiveness were very low in those without comorbidities, without lung disease, not prescribed inhaled corticosteroids, and in NHS risk categories 4, 7, 8, and 9 (ages 70 to 74 years; 60 to 64 years, 55 to 59 years, and 50 to 54 years not at-risk). These results indicate that economic value varies by underlying clinical risk as well as by age (eTables 8 and 9 in Supplement 1).

The apparent nonmonotonic age pattern reflects the use of NHS-defined COVID-19 risk categories rather than author-defined age strata, with ages 70 to 74 years representing a transitional policy group between older adults with substantially higher baseline risk and younger populations where eligibility is driven by clinical vulnerability rather than age alone. Subgroup estimates should therefore be interpreted as exploratory rather than indicative of sharp age-specific thresholds.

Vaccination status further modified cost-effectiveness. Nirmatrelvir-ritonavir was cost-effective with 1 to 3 doses or when the last dose was more than 3 months prior, but not among unvaccinated participants, those vaccinated within 3 months, or with 4 doses. In PANORAMIC, unvaccinated referred to participants who had received no COVID-19 vaccine doses at enrollment. This pattern is clinically plausible. Recent vaccination provides strong protection against severe outcomes, reducing baseline risk and attenuating the absolute benefit and cost-effectiveness of antiviral treatment.^40^ As vaccine-derived protection wanes over time, particularly among older and clinically vulnerable populations, baseline risk may increase and the incremental benefit of nirmatrelvir-ritonavir becomes more pronounced.^2,41^ Vaccination status in this analysis should therefore be interpreted as a proxy for residual protection against severe disease rather than a fixed binary classification, a distinction that is especially relevant in the current UK immunity landscape where unvaccinated may reflect waning protection rather than vaccine naivete.

These findings align with, but also extend, prior economic evaluations of nirmatrelvir-ritonavir conducted across diverse health care systems. Among the 15 economic evaluations of nirmatrelvir-ritonavir identified across diverse health system contexts,^6,7,8,9,10,11,12,13,14,15,16,17,18,19,20^ there is general agreement that the intervention is cost-effective in older adults and those at high clinical risk. However, our within-trial analysis provides a more granular assessment using UK trial data and linked routine health care data and highlights important differences: cost-effectiveness was high in ages 65 to 69 and 75 to 79 years, but not ages 70 to 74 years. This contrasts with Zhang et al,^18^ who reported cost-effectiveness only in those aged over 80 years regardless of vaccination, and Fernandes et al,^15^ who found value mainly in immunosuppressed individuals and those over 60 years. Mizuno et al^7^ reported cost-effectiveness across adults ages 60 years and older, while our results show this does not apply uniformly. Such discrepancies underscore the limitations of coarse age thresholds and support the use of disaggregated age-risk groupings.

Several studies have also emphasized the influence of vaccination status and comorbidities. Savinkina et al^6^ and Wikman-Jorgensen et al^12^ highlighted that nirmatrelvir-ritonavir may offer the most value in unvaccinated or immunosuppressed individuals. Birnie et al^11^ further stressed for the importance of drug price reductions to maintain cost-effectiveness in vaccinated populations. In our highly vaccinated UK setting, we found that vaccination attenuated but did not eliminate the value of antiviral treatment, with cost-effectiveness varying by dose number and recency. Together, these results support a targeted prescribing approach in which nirmatrelvir-ritonavir complements vaccination by providing greatest economic value in individuals with higher residual risk of severe COVID-19.

### Strengths and Limitations

Unlike model-based evaluations from the US and Asia, which typically simulate hospitalizations and QALYs using hypothetical cohorts, our study used empirical data from a large-scale, pragmatic randomized clinical trial with linked routine health records. This strengthens the internal validity of our findings and ensures consistency with UK policy and NICE methodological standards.

However, our study has limitations. As with any pragmatic trial-based economic evaluation, estimates may be subject to information bias from self-reported resource use, residual confounding in subgroup analyses, and potential selection bias due to differential attrition between trial groups, with slightly higher loss to follow-up in the usual care group.

Although subgroup analyses were prespecified, some were based on small sample sizes (under 100 participants per arm). In particular, subgroup analyses involving adults aged 80 years and older, unvaccinated or partially vaccinated participants (including those with zero, 1, or 2 vaccine doses), participants with cardiovascular disease, and the smallest government-defined risk categories (categories 2 and 3) included fewer than 100 participants per group. These subgroup analyses were characterized by limited precision, reflected in wide confidence intervals, and should therefore be interpreted as exploratory and hypothesis-generating rather than providing firm subgroup-specific conclusions. In addition, not all populations prioritized for antiviral treatment in clinical practice were included in PANORAMIC; certain risk groups, such as pregnant women, were excluded and the cost-effectiveness and clinical benefits for these groups remain unknown.

The 6-month within-trial time horizon did not capture longer-term effects, including long COVID, and may underestimate longer-term costs and health consequences, although no clear evidence of differential postacute sequelae has emerged between treatment groups. For analyses adopting a societal perspective, interpretation is further subject to uncertainty in the choice of cost-effectiveness threshold, as no established cost-effectiveness threshold exists to reflect opportunity costs beyond the health care sector.

The study was conducted during a period of high vaccine coverage against COVID-19, particularly among older and clinically at-risk populations, which may limit the applicability of the findings to current and future settings characterized by more heterogeneous immunity from vaccination and prior infection. As a result, the estimated cost-effectiveness reflects a context of relatively reduced baseline risk of severe COVID-19 and may represent a conservative estimate relative to settings with lower levels of preexisting immunity. In addition, the number of unvaccinated participants was very small, limiting the precision of any vaccination-stratified estimates and preclude firm conclusions for unvaccinated individuals. More broadly, the use of evidence from past interventions to inform forward-looking policy scenarios necessarily relies on assumptions about future epidemiological conditions, population immunity, and health system pressures, which may differ from those observed during the trial period.

## Conclusions

Our findings support the cost-effective use of nirmatrelvir-ritonavir in targeted populations, particularly younger adults with comorbidities and older adults aged 75 years and above. Importantly, cost-effectiveness was not observed uniformly across older age groups, nor in low-risk or recently vaccinated populations, challenging a blanket prescribing approach. These results suggest that more refined risk stratification, incorporating age, comorbidity status, and recent vaccination history, should guide future antiviral treatment strategies in the community. Although nirmatrelvir–ritonavir was cost-effective on average, the probability of cost-effectiveness indicates decision uncertainty, highlighting the importance of targeted use and continued evidence generation. While this analysis did not formally assess equity impacts, targeted prescribing may also have important equity implications, as prioritizing groups at higher baseline risk has the potential to reduce absolute health losses. However, implementation of targeted recommendations should consider potential barriers to access to ensure that existing health inequalities are not exacerbated.

## Notes

Supplement 1. eAppendix. PANORAMIC Trial Collaborative Group eTable 1. Unit cost of resources, in 2023/24 prices eTable 2. Baseline characteristics of PANORAMIC participants by treatment group eTable 3. Completion rate of resource use and EQ-5D-5L sections by treatment group eTable 4. Mean (SE) resource use consumption per participant by treatment group at each time-point (available cases) eTable 5. Mean cost (SE) of resource use per participant by treatment arm at each time-point (available cases), in 2023/24 prices eTable 6. Mean (SE) EQ-5D-5L utility and visual analogue scale (VAS) scores by treatment arm at each time-point (available cases) eTable 7. Incremental cost-effectiveness of nirmatrelvir-ritonavir versus usual care over six months from the sensitivity and subgroup analyses, in 2023/24 prices eTable 8. Summary of the cost-effectiveness of nirmatrelvir-ritonavir by age group and risk status under the £20 000 per QALY threshold eTable 9. Summary of the cost-effectiveness of nirmatrelvir-ritonavir by vaccination status under the £20 000 per QALY threshold eReferences.

Supplement 2. Nonauthor Collaborators

Supplement 3. Data Sharing Statement
