# Supplementary material for: Economic Evaluation of Oral Nirmatrelvir-Ritonavir for COVID-19 in Higher Risk Outpatients
Source: JAMA Netw Open. 2026 May 6;9(5):e2612381. doi: 10.1001/jamanetworkopen.2026.12381 (PMC13150634; doi:10.1001/jamanetworkopen.2026.12381)
Supplement: Supplement 1. — eAppendix. PANORAMIC Trial Collaborative Group eTable 1. Unit cost of resources, in 2023/24 prices eTable 2. Baseline characteristics of PANORAMIC participants by treatment group eTable 3. Completion rate of resource use and EQ-5D-5L sections by treatment group eTable 4. Mean (SE) resource use consumption per participant by treatment group at each time-point (available cases) eTable 5. Mean cost (SE) of resource use per participant by treatment arm at each time-point (available cases), in 2023/24 prices eTable 6. Mean (SE) EQ-5D-5L utility and visual analogue scale (VAS) scores by treatment arm at each time-point (available cases) eTable 7. Incremental cost-effectiveness of nirmatrelvir-ritonavir versus usual care over six months from the sensitivity and subgroup analyses, in 2023/24 prices eTable 8. Summary of the cost-effectiveness of nirmatrelvir-ritonavir by age group and risk status under the £20 000 per QALY threshold eTable 9. Summary of the cost-effectiveness of nirmatrelvir-ritonavir by vaccination status under the £20 000 per QALY threshold eReferences. [file jamanetwopen-e2612381-s001.pdf]

Supplemental Online Content

Png ME, Harris V, Yu L, et al; on behalf of the PANORAMIC Trial Collaborative Group. Economic Evaluation of Oral Nirmatrelvir-Ritonavir for COVID-19 in Higher Risk Outpatients. *JAMA Netw Open.* 2026;9(5):e2612381. doi:10.1001/jamanetworkopen.2026.12381

eAppendix. PANORAMIC Trial Collaborative Group

eTable 1. Unit cost of resources, in 2023/24 prices

eTable 2. Baseline characteristics of PANORAMIC participants by treatment group

eTable 3. Completion rate of resource use and EQ-5D-5L sections by treatment group

eTable 4. Mean (SE) resource use consumption per participant by treatment group at each time-point (available cases)

eTable 5. Mean cost (SE) of resource use per participant by treatment arm at each time-point (available cases), in 2023/24 prices

eTable 6. Mean (SE) EQ-5D-5L utility and visual analogue scale (VAS) scores by treatment arm at each time-point (available cases)

eTable 7. Incremental cost-effectiveness of nirmatrelvir-ritonavir versus usual care over six months from the sensitivity and subgroup analyses, in 2023/24 prices

eTable 8. Summary of the cost-effectiveness of nirmatrelvir-ritonavir by age group and risk status under the £20 000 per QALY threshold

eTable 9. Summary of the cost-effectiveness of nirmatrelvir-ritonavir by vaccination status under the £20 000 per QALY threshold

eReferences.

This supplemental material has been provided by the authors to give readers additional information about their work.

PANORAMIC Trial Collaborative Group

Prof Christopher C Butler, FMedSci[1], Prof F D Richard Hobbs, FMedSci[1], Oghenekome A Gbinigie, DPhil[1], Prof Najib M Rahman, DPhil[2,3,5], Gail Hayward, DPhil[1], Prof Duncan B Richards, DM[4], Jienchi Dorward, MBChB[1,6], David M Lowe, PhD[7], Prof Joseph F Standing, PhD[8,9], Prof Judith Breuer, MD[8], Prof Saye Khoo, FRCP[10], Prof Stavros Petrou, PhD[1], Prof Kerenza Hood, PhD[11], Prof Jonathan S Nguyen-Van- Tam, FMedSci[13], Prof Mahendra G Patel, PhD[1], Benjamin R Saville, PhD[14,15], Joe Marion, PhD[14], Prof Nick Francis, PhD[16], Nicholas P B Thomas, FRCGP[17,18,19], Prof Philip Evans, FRCGP[20,21], Melissa Dobson, BSc[2], Jane Holmes, PhD[1], Victoria Harris, PhD[4], May Ee Png, PhD[1], Mark Lown, PhD[16], Oliver van Hecke, DPhil[1], Michelle A Detry, PhD[14], Christina T Saunders, PhD[14], Mark Fitzgerald, PhD[14], Nicholas S Berry, PhD[14], Sam Mort, PGCert[1], Bhautesh D Jani, PhD[22], Prof Nigel D Hart, MD[23], Haroon Ahmed, PhD[12], Daniel Butler, MBChB[23], Micheal McKenna, BSc[1], Lucy Cureton, BSc[1], Meena Patil[1], Monique Andersson, MD[24], Clare Bateman, BA[1], Jennifer C Davies, PhD[1], Prof Andrew Ustianowski, PhD[25], Prof Andrew Carson Stevens, PhD[12], Ly-Mee Yu, DPhil[1], Prof Paul Little, FMedSci[16]

1. Nuffield Department of Primary Care Health Sciences, University of Oxford, Oxford, UK
2. Oxford Respiratory Trials Unit, Nuffield Department of Medicine, University of Oxford, Oxford, UK
3. Chinese Academy of Medical Sciences Oxford Institute, University of Oxford, Oxford, UK
4. Nuffield Department of Orthopaedics, Rheumatology and Musculoskeletal Sciences, University of Oxford, Oxford, UK
5. Oxford National Institute for Health and Care Research Biomedical Research Centre, Oxford, UK
6. Centre for the AIDS Programme of Research in South Africa (CAPRISA), University of KwaZulu–Natal, Durban, South Africa
7. Institute of Immunity and Transplantation, University College London, London, UK
8. Infection, Inflammation and Immunology, UCL Great Ormond Street Institute of Child Health, London, UK
9. Department of Pharmacy, Great Ormond Street Hospital for Children, London, UK
10. Department of Pharmacology, University of Liverpool, Liverpool, UK
11. Centre for Trials Research, Cardiff University, Cardiff, UK
12. Division of Population Medicine, Cardiff University, Cardiff, UK
13. Lifespan and Population Health Unit, University of Nottingham School of Medicine, Nottingham, UK
14. Berry Consultants, Austin, TX, USA
15. Department of Biostatistics, Vanderbilt School of Medicine, Nashville, TN, USA
16. Primary Care Research Centre, University of Southampton, Southampton, UK
17. Windrush Medical Practice, Witney, UK
18. National Institute for Health and Care Research Clinical Research Network: Thames Valley and South Midlands, Oxford, UK
19. Royal College of General Practitioners, London, UK
20. Faculty of Health and Life Sciences, University of Exeter, Exeter, UK
21. National Institute for Health and Care Research Clinical Research Network, Leeds, UK
22. General Practice and Primary Care, School of Health and Wellbeing, College of Medical, Veterinary & Life Sciences, University of Glasgow, Glasgow, UK
23. School of Medicine, Dentistry and Biomedical Sciences, Queen's University Belfast, Belfast, UK
24. Department of Microbiology, Oxford University Hospitals NHS Foundation Trust, Oxford, UK
25. Regional Infectious Diseases Unit, North Manchester General Hospital, Manchester, UK

Clinical safety call team

Debby Nicoll, Adrian Burns, Florence Conneh, Leon Dong, Mary Green, Bhumika Patel, Amrita Kafle, Andrew Ting, Haroen Sahak, Juliah Jonasi, Wahida Kayyum, Sanjay Ramakrishnan, Rita Corser,

© 2026 Png ME et al. *JAMA Network Open*.

Tsvetely Angelova-Cooper, Bindhu Xavier, Darren Smith, Dawn Beaumont-Jewell, Robin Williams, Kerry Goodsell, Samantha Edwards, Chisomo Chitedze, Karolina Krassowska, Jess Trigg

Non-clinical safety call team

Nicholas Symons, Suzie Engela Amber, Madden-Nadeau, Faith Fordham

Clinical safety monitor

Usha Sukumaran, Areej Moftah

Clinical safety monitor (rota cover)

Liliana Cifuentes Gutierrez, Kome Gbinigie, Monique Andersson, Najib Rahman, Chris Turnbull, Rob Hallifax, Anand Sundaralingam

Independent SAE reviewers

Mark Lown, Beenish Iqbal, Dinesh Addala, Mark Roberts, Peter Saunders

Virology team

Akosua A Agyeman, Divya Shah, Julianne Brown, Chris Thalasselis, Maximillian N J Woodall, Francis Yongblah

Principal investigators and Associate Principal Investigators

Tanveer Ahmed, Aleksandra Howell, Kavil Patel, Damien Allcock, Iqbal Hussain, Ruth Penfold, George Atherton, Simon Hutchinson, Satveer Poonian, Oluseye Emmanuel Benedict, Marie Imlach, Olajide Popoola, Adrian Beltran-Martinez, Greg Irving, Alexander Pora, Nigel Bird, Nicholas Jacobsen, Vibhore Prasad, Gerard Burns, James Kennard, Rishabh Prasad, Laura Brennan, Umar Khan, Omair Razzaq, Mike Butler, Kyle Knox, Scot Richardson, Daniel Butler, Christopher Krasucki, Simon Royal, Andrew Carson-Stevens, Tom Law, Afsana Safa, Zelda Cheng, Rem Lee, Satash Sehdev, Ruth Danson, Nicola Lester, Tamsin Sevenoaks, Nigel de Kare-Silver, David Lewis, Aadil Sheikh, Devesh Dhasmana, James Lunn, Vanessa Short, Jon Dickson, Claire I. Mackintosh, Baljinder Singh Sidhu, Serge Engamba, Mehul Mathukia, Ivor Singh, Stacey Fisher, Patrick Moore, Yusuf Soni, Robin Fox, Seb Morton, Pete Wilson, Eve Frost, Daniel Murphy, David Wingfield, Richard Gaunt, Rhiannon Nally, Michael Wong, Sarit Ghosh, Chinonso Ndukauba, Nick Wooding, Ishtiaq Gilkar, Olufunto Ogundapo, Sharon Woods, Anna Goodman, Henry Okeke, Joanna Yong, Steve Granier, Amit Patel, Azhar Zafar

Data and Safety Monitoring Committee Independent members

Prof Deborah Ashby (Chair), Prof Simon Gates, Prof Benjamin Fisher, Prof Gordon Taylor, Prof Martin Underwood

Trial Steering Committee Independent members

Prof Philip Hannaford (Chair), Ms Corina Cheeks, Prof Ranjit Lall, Prof Alastair Hay, Prof William Hollingworth

© 2026 Png ME et al. *JAMA Network Open*.

TABLES

eTable 1. Unit cost of resources, in 2023/24 prices

Resource item	Unit	Unit cost, £	Reference
Treatment			
Nirmatrelvir-ritonavir	per 5-day course	829	BNF 2025[1]
Health services			
Accident & emergency	per contact	189	NCC 2023/24[2]
Abdominal aortic aneurysm screening*	per procedure	36.07	Multicentre Aneurysm Screening Study Group 2002[3]
Allergy clinic	per contact	249	NCC 2023/24[2]
Ambulance	per use	327	NCC 2023/24[2]
Anticoagulation clinic	per contact	71	NCC 2023/24[2]
Audiology clinic	per contact	159	NCC 2023/24[2]
Band 2 professional	per contact	1.91	NHS Employers 2023/24[4]
Band 2 professional phone consult	per call	1.79	NHS Employers 2023/24[4]
Band 3 professional	per contact	2.075	NHS Employers 2023/24[4]
Band 3 professional phone consult	per call	1.95	NHS Employers 2023/24[4]
Band 4 professional	per contact	6.5	PSSRU 2024[5]
Band 5 community nurse	per contact	8.83	PSSRU 2024[5]
Band 5 community nurse home consult	per contact	10.07	PSSRU 2024[5]
Band 5 community nurse phone consult	per call	8.30	PSSRU 2024[5]
Band 5 community professional	per contact	7.33	PSSRU 2024[5]
Band 5 community professional phone consult	per call	6.89	PSSRU 2024[5]
Band 5 hospital nurse phone consult	per call	8.15	PSSRU 2024[5]
Band 6 community professional	per contact	9.50	PSSRU 2024[5]
Band 6 community professional home consult	per contact	10.83	PSSRU 2024[5]
Band 6 community professional phone consult	per call	8.93	PSSRU 2024[5]
Band 6 hospital professional phone consult	per call	8.62	PSSRU 2024[5]
Band 7 community nurse	per contact	12.50	PSSRU 2024[5]
Band 7 community professional	per contact	11.33	PSSRU 2024[5]
Blood test	per procedure	9	NCC 2023/24[2]
Bluecrest private health check	per contact	149	Bluecrest 2023[6]
Bone density scan	per procedure	210	NCC 2023/24[2]
Breast surgery clinic	per contact	210	NCC 2023/24[2]
Cardiac rehabilitation clinic	per contact	115	NCC 2023/24[2]
Cardiology clinic	per contact	168	NCC 2023/24[2]
Cardiothoracic clinic	per contact	265	NCC 2023/24[2]
Chiropractor	per contact	50	NHS 2023[7]
Clinical massage	per contact	60	Lymphoma Association 2024[8]
Covid medicines delivery unit (CMDU)	per contact	427.67	NCC 2023/24[2]
Colonoscopy	per procedure	1234	NCC 2023/24[2]
Colorectal clinic	per contact	148	NCC 2023/24[2]
Contact carer home contact	per contact	5.60	Local Government Association 2024[9]
Council	per contact	1.97	British Council[10]
Council phone contact	per call	1.86	British Council[10]
Counsellor	per contact	52.25	PSSRU 2024[5]
Computerized tomography scan	per procedure	109	NCC 2023/24[2]
Cystoscopy	per procedure	258	NCC 2023/24[2]
Dental clinic	per contact	160	NCC 2023/24[2]
Dermatology clinic	per contact	158	NCC 2023/24[2]
Diabetes clinic	per contact	204	NCC 2023/24[2]
Diabetes eye screening	per procedure	42.15	NCC 2023/24[2]
Dietetics	per contact	103	NCC 2023/24[2]
District nurse*	per contact	10.52	PSSRU 2015[11]
Doppler scan	per procedure	80	NCC 2023/24[2]
Electrocardiography	per procedure	176	NCC 2023/24[2]
Echocardiography	per procedure	108	NCC 2023/24[2]
E-consult	per contact	45.98	PSSRU 2024[5]
Electroencephalogram	per procedure	312	NCC 2023/24[2]
Endobronchial ultrasound	per procedure	894	NCC 2023/24[2]

© 2026 Png ME et al. *JAMA Network Open*.

Resource item	Unit	Unit cost, £	Reference
Endocrinology clinic	per contact	205	NCC 2023/24[2]
Endoscopy	per procedure	139	NCC 2023/24[2]
Ear, Nose and Throat clinic	per contact	163	NCC 2023/24[2]
E-prescription	per contact	9.9	NHS 2024[12]
Eye clinic	per contact	140	NCC 2023/24[2]
Gastroenterology clinic	per contact	161	NCC 2023/24[2]
Gastroscopy	per procedure	741	NCC 2023/24[2]
Genetic clinic	per contact	533	NCC 2023/24[2]
Geriatric clinic	per contact	367	NCC 2023/24[2]
General practitioner	per contact	45	PSSRU 2024[5]
General practitioner phone consult	per call	31.71	PSSRU 2024[5]
Gynaecology clinic	per contact	196	NCC 2023/24[2]
Haematology clinic	per contact	184	NCC 2023/24[2]
Health trainer	per contact	4.15	NHS Employers 2023/24[4]
Heart and chest department	per contact	265	NCC 2023/24[2]
Hepatology clinic	per contact	190	NCC 2023/24[2]
Herbalist	per contact	82.5	Prospects 2024[13]
HIV clinic	per contact	193	NCC 2023/24[2]
Home carer	per contact	13.5	PSSRU 2024[5]
Home carer phone contact	per call	4.23	PSSRU 2024[5]
Hospital pharmacist	per contact	282.29	PSSRU 2024[5]
Hyperbaric oxygen therapy	per procedure	226	NCC 2023/24[2]
Immunology clinic	per contact	396	NCC 2023/24[2]
Intravenous drip	per procedure	399	NCC 2023/24[2]
Learning disability team	per contact	119	PSSRU 2024[5]
Mammogram*	per procedure	61.69	NHS Reference Costs 2005/06[14]
Medical consultant phone consult	per call	12.19	PSSRU 2024[5]
Mental health unit	per contact	678	NCC 2023/24[2]
Magnetic resonance imaging	per procedure	156	NCC 2023/24[2]
Muscle test	per procedure	243	NCC 2023/24[2]
Nephrology clinic	per contact	202	NCC 2023/24[2]
Neurology clinic	per contact	235	NCC 2023/24[2]
Neurosurgery clinic	per contact	238	NCC 2023/24[2]
NHS 111/119/999	per call	22	NCC 2023/24[2]
Nuclear stress test	per procedure	403	NCC 2023/24[2]
Occupational therapist	per contact	20.70	PSSRU 2024[5]
Oncology clinic	per contact	193	NCC 2023/24[2]
Ophthalmology clinic	per contact	140	NCC 2023/24[2]
Oral surgery clinic	per contact	203	NCC 2023/24[2]
Orthopaedic clinic	per contact	166	NCC 2023/24[2]
Orthoptic clinic	per contact	102	NCC 2023/24[2]
Orthotic clinic	per contact	188	NCC 2023/24[2]
Osteopath	per contact	33	PSSRU 2024[5]
Pain clinic	per contact	228	NCC 2023/24 [2]
Palliative medicine	per contact	243	NCC 2023/24 [2]
Parathyroid scan	per procedure	460	NCC 2023/24 [2]
Pathology test	per procedure	2	NCC 2023/24 [2]
Positron emission tomography scan	per procedure	615	NCC 2023/24 [2]
Physiotherapist	per contact	44	PSSRU 2024[5]
Podiatry clinic	per contact	103	NCC 2023/24[2]
Post COVID-19 clinic	per contact	288	NCC 2023/24[2]
Practice nurse	per contact	8.83	PSSRU 2024[5]
Practice nurse phone consult	per call	8.30	PSSRU 2024[5]
Psychiatry clinic	per contact	282	NCC 2023/24[2]
Psychology clinic	per contact	281	NCC 2023/24[2]
Pulmonary function test	per procedure	236	NCC 2023/24[2]
Pulmonary rehabilitation clinic	per contact	74	NCC 2023/24[2]
Radiology clinic	per contact	39	NCC 2023/24[2]
Rapid response team*	per contact	7.67	PSSRU 2013[15]
Reablement service*	per contact	9.22	Glendinning 2010[16]
Rehabilitation	per contact	159	NCC 2023/24[2]
Renal clinic	per contact	202	NCC 2023/24[2]
Respiratory clinic	per contact	196	NCC 2023/24[2]

© 2026 Png ME et al. *JAMA Network Open*.

Resource item	Unit	Unit cost, £	Reference
Rheumatology clinic	per contact	181	NCC 2023/24[2]
Sexual health clinic	per contact	177	NCC 2023/24[2]
Small Intestinal Bacterial Overgrowth (SIBO) test	per procedure	2	NCC 2023/24[2]
Sigmoidoscopy	per procedure	498	NCC 2023/24[2]
Sleep clinic	per contact	112	NCC 2023/24[2]
Smoking cessation	per contact	257	NCC 2023/24[2]
Social worker	per contact	29	PSSRU 2024[5]
Social worker phone consult	per call	9.09	PSSRU 2024[5]
Speech and language therapy	per contact	235	NCC 2023/24[2]
Sports injury clinic	per contact	215	NCC 2023/24[2]
Stool test*	per procedure	6.72	Murphy 2017[17]
Stroke clinic	per contact	313	NCC 2023/24[2]
Thoracic surgery clinic	per contact	218	NCC 2023/24[2]
Transient ischaemic attack clinic	per contact	296	NCC 2023/24[2]
Transplant team	per contact	348	NCC 2023/24[2]
Ultrasound	per procedure	61	NCC 2023/24[2]
Urgent care centre	per contact	124	NCC 2023/24[2]
Urology clinic	per contact	152	NCC 2023/24[2]
Vascular clinic	per contact	197	NCC 2023/24[2]
Virtual ward/ hospital-at-home*	per call	28.43	Health Innovation Network 2021[18]
Wellness clinic*	per contact	44.79	PSSRU 2020[19]
X-ray	per procedure	101	NCC 2023/24[2]
Median wage			
All	per day	122.66	ONS 2024[20]
Female	per day	119.03	ONS 2024[21]
Male	per day	136.95	ONS 2024[21]

* Prices have been adjusted to 2023/24£ prices using the Hospital & Community Health Services (HCHS) index.

BNF: British National Formulary, NCC: National Cost Collection, ONS: Office for National Statistics, PSSRU: Personal Social Services Resource Unit. Band 2 professionals include healthcare assistant, hospital call handler and care navigation; Band 3 professionals include receptionist, consultant secretary, and hospital patient liaison; Band 4 professionals include assistant practitioner and hearing care technician; Band 5 professionals include acupuncturist, dentist, dietitian, general practitioner manager, mental health coordinator, podiatrist, nutritionist, orthoptist, paramedic, podiatrist, and radiologist; Band 6 professionals include occupational health advisor, medical care support worker, ophthalmologist, optician, optometrist, psychiatrist, pharmacist and psychotherapist.

© 2026 Png ME et al. *JAMA Network Open*.

eTable 2. Baseline characteristics of PANORAMIC participants by treatment group

	Nirmatrelvir-ritonavir (n=1736)	Usual care (n=1768)
Mean age, years (SE)	54.7 (0.29)	54.8 (0.28)
Mean baseline EQ-5D-5L score (SE)	0.675 (0.0054)	0.682 (0.0052)
Mean baseline EQ-5D-5L VAS (SE)	50.5 (0.47)	50.5 (0.47)
Country (%)		
England	1584 (91.2)	1614 (91.3)
Wales	64 (3.7)	58 (3.3)
Scotland	79 (4.6)	81 (4.6)
Northern Ireland	9 (0.5)	14 (0.8)
Female (%)	1182 (68.1)	1223 (69.2)
Ethnicity (%)		
White	1647 (94.9)	1661 (93.9)
White and Black Caribbean	7 (0.4)	9 (0.5)
White and Black African	0 (0.0)	3 (0.2)
White and Asian	14 (0.8)	6 (0.3)
Caribbean	3 (0.2)	3 (0.2)
African	4 (0.2)	4 (0.2)
Any other Black/African/Caribbean background	1 (0.1)	2 (0.1)
Indian	17 (1.0)	19 (1.1)
Pakistani	4 (0.2)	3 (0.2)
Bangladeshi	2 (0.1)	1 (0.1)
Arab	0 (0.0)	2 (0.1)
Chinese	7 (0.4)	7 (0.4)
Any other Asian background	6 (0.3)	15 (0.8)
Any other Mixed/Multiple ethnic background	13 (0.7)	23 (1.3)
Other	11 (0.6)	10 (0.6)
Non-smoker (%)	1616 (93.1)	1652 (93.4)
Vulnerability (%)		
Respiratory disease	454 (26.2)	439 (24.8)
Cardiovascular disease	83 (4.8)	63 (3.6)
Kidney disease	9 (0.5)	7 (0.4)
Liver disease	17 (1.0)	17 (1.0)
Neurological disease	60 (3.5)	66 (3.7)
Learning disability	8 (0.5)	8 (0.5)
Down's syndrome	6 (0.3)	5 (0.3)
Diabetes mellitus	132 (7.6)	150 (8.5)
Immune disorders	152 (8.8)	166 (9.4)
Transplant recipient	11 (0.6)	7 (0.4)
Obesity	293 (16.9)	312 (17.6)
Mental illness	32 (1.8)	25 (1.4)
Hypertension	221 (12.7)	253 (14.3)
Other	329 (19.0)	349 (19.7)
Comorbidity (%)	1127 (64.9)	1185 (67.0)
Has major symptoms at baseline (%)	749 (43.1)	723 (40.9)
Duration of symptoms at baseline (%)		

© 2026 Png ME et al. *JAMA Network Open*.

	Nirmatrelvir-ritonavir (n=1736)	Usual care (n=1768)
3 or less days	1262 (72.7)	1299 (73.5)
More than 3 days	474 (27.3)	468 (26.5)
Use of inhaled corticosteroid (%)	468 (27.0)	462 (26.1)
Vaccination status (%)		
Not vaccinated	21 (1.2)	28 (1.6)
Vaccinated	1715 (98.8)	1740 (98.4)
No. of dose of vaccination (%)		
0	21 (1.2)	28 (1.6)
1	16 (0.9)	7 (0.4)
2	78 (4.5)	84 (4.8)
3	656 (37.8)	693 (39.2)
4+	723 (41.6)	704 (39.8)
Time elapsed since most recent dose (%)		
Not vaccinated	21 (1.2)	28 (1.6)
0 to 3 months	320 (18.4)	295 (16.7)
3 to 6 months	353 (20.3)	366 (20.7)
More than 6 months	978 (56.3)	1004 (56.8)
Swab positivity status (%)		
PCR positive	584 (33.6)	629 (35.6)
LFD positive	1152 (66.4)	1139 (64.4)
Risk category as defined by NHS (%)		
2	16 (0.9)	13 (0.7)
3	51 (2.9)	42 (2.4)
4	113 (6.5)	121 (6.8)
5	156 (9.0)	174 (9.8)
6	940 (54.1)	978 (55.3)
7	125 (7.2)	100 (5.7)
8	165 (9.5)	179 (10.1)
9	170 (9.8)	161 (9.1)

© 2026 Png ME et al. *JAMA Network Open*.

eTable 3. Completion rate of resource use and EQ-5D-5L sections by treatment group

	**Nirmatrelvir-ritonavir (n=1736)**	**Usual care (n=1768)**
Resource use*, n (%)		
First 28 days	1697 (97.8)	1685 (95.3)
28 days to 3 months	1610 (92.7)	1638 (92.6)
3 months to 6 months	1591 (91.6)	1614 (91.3)
EQ-5D-5L, n (%)		
Baseline	1736 (100.0)	1768 (100.0)
Baseline to 14 days	1550 (89.3)	1445 (81.7)
14 days to 28 days	1505 (86.7)	1428 (80.8)
28 days to 3 months	1484 (85.5)	1414 (80.0)
3 months to 6 months	1459 (84.0)	1403 (79.4)

*Collected from both trial and routine data

© 2026 Png ME et al. *JAMA Network Open*.

eTable 4. Mean (SE) resource use consumption per participant by treatment group at each time-point (available cases)

Resource category	Unit	Nirmatrelvir-ritonavir (n=1736)	Usual care (n=1768)	Mean difference	p-value*	Bootstrap 95%CI
**First 28 days**		**n=1697**	**n=1685**			
Admitted patient care	per episode	0.0165 (0.0031)	0.0231 (0.0044)	-0.0066	0.22	-0.0173 to 0.0035
Critical care	per episode	0.0000 (0.0000)	0.0000 (0.0000)	0.0000	NA	NA
Accident & emergency	per visit	0.0854 (0.0077)	0.0866 (0.0085)	-0.0012	0.92	-0.0237 to 0.0207
Respiratory outpatient	per visit	0.2239 (0.0147)	0.2487 (0.0157)	-0.0247	0.25	-0.0679 to 0.0178
NHS 111	per call	0.0648 (0.0076)	0.1068 (0.0100)	-0.0420	0.00083	-0.0669 to -0.0177
Ambulance	per use	0.0260 (0.0041)	0.0328 (0.0056)	-0.0068	0.33	-0.0211 to 0.0065
GP	per contact	0.4390 (0.0276)	0.4777 (0.0255)	-0.0387	0.3	-0.1118 to 0.0350
Community nurse	per contact	0.0337 (0.0083)	0.0273 (0.0049)	0.0064	0.51	-0.0114 to 0.0266
Physiotherapist	per contact	0.0112 (0.0037)	0.0170 (0.0053)	-0.0058	0.37	-0.0190 to 0.0062
Counsellor	per contact	0.0249 (0.0117)	0.0182 (0.0059)	0.0066	0.61	-0.0160 to 0.0352
Social worker	per contact	0.0024 (0.0012)	0.0049 (0.0032)	-0.0025	0.47	-0.0104 to 0.0029
Home carer	per contact	0.0367 (0.0193)	0.0437 (0.0228)	-0.0071	0.81	-0.0666 to 0.0502
Occupational therapist	per contact	0.0183 (0.0033)	0.0255 (0.0044)	-0.0072	0.19	-0.0180 to 0.0037
Hospital at home for COVID-19	per contact	0.0343 (0.0067)	0.0990 (0.0311)	-0.0647	0.042	-0.1329 to -0.0100
Primary care prescriptions	per prescription	0.4985 (0.0139)	0.5792 (0.0133)	-0.0807	0.000027	-0.1180 to -0.0428
Others	per contact	0.0266 (0.0061)	0.0571 (0.0097)	-0.0305	0.0078	-0.0533 to -0.0094
**28 days to 3 months**		**n=1610**	**n=1638**			
Admitted patient care	per episode	0.0932 (0.0098)	0.0977 (0.0100)	-0.0045	0.75	-0.0329 to 0.0221
Critical care	per episode	0.0012 (0.0009)	0.0018 (0.0011)	-0.0006	0.67	-0.0031 to 0.0019
Accident & emergency	per visit	0.1273 (0.0119)	0.1105 (0.0121)	0.0168	0.32	-0.0170 to 0.0493
Respiratory outpatient	per visit	0.7155 (0.0370)	0.7692 (0.0392)	-0.0537	0.32	-0.1595 to 0.0510
NHS 111	per call	0.0360 (0.0111)	0.0275 (0.0069)	0.0086	0.51	-0.0149 to 0.0370
Ambulance	per use	0.0020 (0.0012)	0.0014 (0.0010)	0.0006	0.69	-0.0022 to 0.0034
GP surgery	per contact	0.1348 (0.0185)	0.1154 (0.0124)	0.0194	0.38	-0.0228 to 0.0644
GP home visit	per contact	0.0006 (0.0006)	0.0000 (0.0000)	0.0006	0.32	0.0000 to 0.0019
GP phone consult	per contact	0.2149 (0.0239)	0.1905 (0.0198)	0.0244	0.43	-0.0349 to 0.0850
GP video consult	per contact	0.0141 (0.0075)	0.0098 (0.0046)	0.0043	0.63	-0.0115 to 0.0231
Practice nurse	per contact	0.0483 (0.0069)	0.0714 (0.0118)	-0.0231	0.092	-0.0505 to 0.0026
Community nurse	per contact	0.0101 (0.0065)	0.0007 (0.0007)	0.0094	0.15	-0.0001 to 0.0248

© 2026 Png ME et al. *JAMA Network Open*.

Resource category	Unit	Nirmatrelvir-ritonavir (n=1736)	Usual care (n=1768)	Mean difference	p-value*	Bootstrap 95%CI
Physiotherapist	per contact	0.0335 (0.0088)	0.0308 (0.0079)	0.0027	0.82	-0.0204 to 0.0263
Counsellor	per contact	0.0282 (0.0100)	0.0623 (0.0305)	-0.0341	0.29	-0.1072 to 0.0171
Social worker	per contact	0.0013 (0.0013)	0.0007 (0.0007)	0.0006	0.67	-0.0021 to 0.0040
Home carer	per contact	0.0637 (0.0542)	0.0000 (0.0000)	0.0637	0.24	0.0007 to 0.1838
Occupational therapist	per contact	0.0101 (0.0038)	0.0294 (0.0180)	-0.0193	0.29	-0.0628 to 0.0064
Primary care prescriptions	per prescription	1.7398 (0.0383)	1.8608 (0.0343)	-0.1211	0.019	-0.2211 to -0.0206
Time off work**	per day	3.7827 (0.3490)	4.2176 (0.3740)	-0.4349	0.400	-1.4467 to 0.5662
Others	per contact	0.0463 (0.0130)	0.0469 (0.0092)	-0.0006	0.97	-0.0301 to 0.0327
**3 months to 6 months**		**n=1591**	**n=1614**			
Admitted patient care	per episode	0.0710 (0.0087)	0.0836 (0.0085)	-0.0126	0.30	-0.0363 to 0.0117
Critical care	per episode	0.0006 (0.0006)	0.0006 (0.0006)	0.0000	0.99	-0.0019 to 0.0019
Accident & emergency	per visit	0.1043 (0.0235)	0.0942 (0.0092)	0.0102	0.69	-0.0300 to 0.0649
Respiratory outpatient	per visit	0.4991 (0.0286)	0.5297 (0.0280)	-0.0307	0.44	-0.1089 to 0.0465
NHS 111	per call	0.0226 (0.0059)	0.0248 (0.0056)	-0.0022	0.79	-0.0178 to 0.0141
Ambulance	per use	0.0034 (0.0015)	0.0035 (0.0021)	-0.0001	0.96	-0.0057 to 0.0047
GP surgery	per contact	0.1288 (0.0137)	0.1450 (0.0163)	-0.0161	0.45	-0.0573 to 0.0245
GP home visit	per contact	0.0000 (0.0000)	0.0012 (0.0009)	-0.0012	0.16	-0.0031 to 0.0000
GP phone consult	per contact	0.1999 (0.0248)	0.1952 (0.0234)	0.0047	0.89	-0.0630 to 0.0720
GP video consult	per contact	0.0095 (0.0058)	0.0028 (0.0014)	0.0067	0.26	-0.0022 to 0.0209
Practice nurse	per contact	0.0639 (0.0099)	0.0680 (0.0148)	-0.0040	0.82	-0.0405 to 0.0287
Community nurse	per contact	0.0054 (0.0054)	0.0021 (0.0021)	0.0033	0.57	-0.0064 to 0.0163
Physiotherapist	per contact	0.0388 (0.0107)	0.0588 (0.0157)	-0.0200	0.29	-0.0571 to 0.0161
Counsellor	per contact	0.0422 (0.0147)	0.0779 (0.0329)	-0.0357	0.32	-0.1126 to 0.0262
Social worker	per contact	0.0116 (0.0085)	0.0014 (0.0010)	0.0101	0.24	-0.0015 to 0.0299
Home carer	per contact	0.1259 (0.1225)	0.0064 (0.0057)	0.1195	0.33	-0.0127 to 0.3734
Occupational therapist	per contact	0.0286 (0.0101)	0.0269 (0.0098)	0.0017	0.91	-0.0266 to 0.0291
Primary care prescriptions	per prescription	1.1307 (0.0262)	1.2069 (0.0225)	-0.0762	0.028	-0.1440 to -0.0077
Time off work	per day	3.5898 (0.5763)	2.9122 (0.4935)	0.6776	0.37	-0.8228 to 2.1888
Others	per contact	0.0361 (0.0100)	0.0765 (0.0277)	-0.0404	0.17	-0.1069 to 0.0073

*Obtained from t-test

**Time-off work in the 3-month questionnaire was measured from baseline to 3 months

© 2026 Png ME et al. *JAMA Network Open*.

eTable 5. Mean cost (SE) of resource use per participant by treatment arm at each time-point (available cases), in 2023/24 prices

Resource category	Unit	Nirmatrelvir-ritonavir (n=1736)	Usual care (n=1768)	Mean difference	p-value*	Bootstrap 95%CI
**First 28 days**		**n=1697**	**n=1685**			
Admitted patient care	per episode	55.54 (19.18)	157.30 (68.73)	-101.76	0.15	-261 to 17
Critical care	per episode	0.00 (0.000)	0.00 (0.000)	0.000	NA	NA
Accident & emergency	per visit	20.18 (2.072)	19.01 (2.129)	1.170	0.69	-4.77 to 6.88
Respiratory outpatient	per visit	3.75 (0.772)	4.87 (1.038)	-1.123	0.39	-3.76 to 1.38
NHS 111	per call	1.43 (0.168)	2.35 (0.220)	-0.924	0.0008	-1.47 to -0.39
Ambulance	per use	8.51 (1.325)	10.73 (1.845)	-2.214	0.33	-6.90 to 2.14
GP	per contact	19.76 (1.240)	21.50 (1.147)	-1.743	0.30	-5.03 to 1.58
Community nurse	per contact	0.30 (0.073)	0.24 (0.043)	0.056	0.51	-0.10 to 0.23
Physiotherapist	per contact	0.49 (0.162)	0.75 (0.232)	-0.254	0.37	-0.84 to 0.27
Counsellor	per contact	1.30 (0.611)	0.95 (0.307)	0.346	0.61	-0.84 to 1.84
Social worker	per contact	0.07 (0.034)	0.14 (0.093)	-0.072	0.47	-0.30 to 0.09
Home carer	per contact	0.50 (0.261)	0.59 (0.307)	-0.095	0.81	-0.90 to 0.68
Occupational therapist	per contact	0.38 (0.068)	0.53 (0.092)	-0.148	0.19	-0.37 to 0.08
Hospital at home for COVID-19	per contact	0.98 (0.192)	2.82 (0.883)	-1.840	0.042	-3.78 to -0.29
Primary care prescriptions	per prescription	16.45 (0.968)	19.93 (1.075)	-3.486	0.016	-6.30 to -0.70
Others	per contact	1.93 (0.697)	6.45 (1.164)	-4.527	0.0009	-7.17 to -1.91
**28 days to 3 months**		**n=1610**	**n=1638**			
Admitted patient care	per episode	576.53 (199.73)	444.01 (118.17)	132.52	0.57	-274 to 624
Critical care	per episode	8.60 (6.683)	7.84 (4.562)	0.760	0.93	-13.84 to 18.04
Accident & emergency	per visit	31.21 (3.172)	27.84 (3.654)	3.369	0.49	-6.23 to 12.65
Respiratory outpatient	per visit	4.81 (1.210)	4.72 (1.550)	0.089	0.96	-4.01 to 3.80
NHS 111	per call	0.79 (0.244)	0.60 (0.152)	0.188	0.51	-0.33 to 0.81
Ambulance	per use	0.66 (0.380)	0.46 (0.324)	0.200	0.69	-0.71 to 1.10
GP surgery	per contact	6.07 (0.831)	5.19 (0.556)	0.873	0.38	-1.03 to 2.90
GP home visit	per contact	0.03 (0.032)	0.00 (0.000)	0.032	0.32	0.00 to 0.09
GP phone consult	per contact	6.82 (0.759)	6.04 (0.627)	0.775	0.43	-1.11 to 2.69
GP video consult	per contact	0.65 (0.344)	0.45 (0.213)	0.197	0.63	-0.53 to 1.06
Practice nurse	per contact	0.43 (0.061)	0.63 (0.104)	-0.204	0.092	-0.45 to 0.02
Community nurse	per contact	0.09 (0.058)	0.01 (0.006)	0.083	0.15	-0.00051 to 0.22
Physiotherapist	per contact	1.48 (0.388)	1.35 (0.349)	0.121	0.82	-0.90 to 1.16
Counsellor	per contact	1.47 (0.523)	3.25 (1.594)	-1.782	0.29	-5.60 to 0.89
Social worker	per contact	0.04 (0.039)	0.02 (0.020)	0.019	0.67	-0.061 to 0.12
Home carer	per contact	0.86 (0.732)	0.00 (0.000)	0.860	0.24	0.0091 to 2.48
Occupational therapist	per contact	0.21 (0.080)	0.61 (0.373)	-0.400	0.29	-1.30 to 0.13
Primary care prescriptions	per prescription	61.66 (4.244)	67.03 (3.072)	-5.374	0.31	-15.15 to 5.48
Time off work**	per day	464.25 (42.965)	520.23 (46.391)	-55.977	0.38	-181 to 67
Others	per contact	3.52 (0.963)	6.99 (1.511)	-3.472	0.05	-7.04 to 0.00082
**3 months to 6 months**		n=1591	n=1614			
Admitted patient care	per episode	210.69 (72.428)	489.55 (128.964)	-278.858	0.06	-589 to -7.3
Critical care	per episode	2.40 (2.397)	1.17 (1.168)	1.229	0.64	-3.50 to 7.19

© 2026 Png ME et al. *JAMA Network Open*.

Resource category	Unit	Nirmatrelvir-ritonavir (n=1736)	Usual care (n=1768)	Mean difference	p-value*	Bootstrap 95%CI
Accident & emergency	per visit	23.63 (4.749)	25.31 (2.804)	-1.679	0.76	-11.31 to 9.87
Respiratory outpatient	per visit	5.79 (1.375)	4.72 (1.049)	1.072	0.54	-2.24 to 4.60
NHS 111	per call	0.50 (0.131)	0.55 (0.124)	-0.047	0.79	-0.39 to 0.31
Ambulance	per use	1.11 (0.497)	1.16 (0.694)	-0.046	0.96	-1.86 to 1.54
GP surgery	per contact	5.80 (0.616)	6.52 (0.734)	-0.726	0.45	-2.58 to 1.10
GP home visit	per contact	0.00 (0.000)	0.06 (0.045)	-0.063	0.16	-0.16 to 0.00
GP phone consult	per contact	6.34 (0.787)	6.19 (0.742)	0.149	0.89	-2.00 to 2.28
GP video consult	per contact	0.44 (0.265)	0.13 (0.065)	0.308	0.26	-0.10 to 0.96
Practice nurse	per contact	0.56 (0.088)	0.60 (0.130)	-0.036	0.82	-0.36 to 0.25
Community nurse	per contact	0.05 (0.048)	0.02 (0.019)	0.029	0.57	-0.06 to 0.14
Physiotherapist	per contact	1.71 (0.472)	2.59 (0.690)	-0.880	0.29	-2.51 to 0.71
Counsellor	per contact	2.20 (0.767)	4.07 (1.717)	-1.867	0.32	-5.88 to 1.37
Social worker	per contact	0.34 (0.247)	0.04 (0.029)	0.294	0.24	-0.04 to 0.87
Home carer	per contact	1.70 (1.654)	0.09 (0.077)	1.613	0.33	-0.17 to 5.04
Occupational therapist	per contact	0.59 (0.209)	0.56 (0.204)	0.034	0.91	-0.55 to 0.60
Primary care prescriptions	per prescription	39.39 (2.052)	43.35 (2.077)	-3.959	0.18	-9.66 to 1.81
Time off work	per day	444.60 (71.655)	353.49 (59.597)	91.113	0.33	-93 to 275
Others	per contact	4.49 (1.144)	8.87 (2.008)	-4.378	0.058	-9.08 to -0.0064

*Obtained from t-test

**Time-off work in the 3-month questionnaire was measured from baseline to 3 months

© 2026 Png ME et al. *JAMA Network Open*.

eTable 6. Mean (SE) EQ-5D-5L utility and visual analogue scale (VAS) scores by treatment arm at each time-point (available cases)

	Nirmatrelvir-ritonavir (n=1736)	Usual care (n=1768)	Mean difference	p-value*	Bootstrap 95%CI
EQ-5D-5L utility					
Baseline	0.6750 (0.0054)	0.6815 (0.0052)	-0.0065	0.39	-0.0213 to 0.0078
14-day	0.8357 (0.0049)	0.8116 (0.0051)	0.0241	<0.0001	0.0103 to 0.0380
28-day	0.8581 (0.0048)	0.8429 (0.0052)	0.0153	0.030	0.0014 to 0.0294
3-month	0.8487 (0.0053)	0.8333 (0.0057)	0.0154	0.048	0.0000 to 0.0303
6-month	0.8439 (0.0055)	0.8365 (0.0053)	0.0074	0.34	-0.0079 to 0.0224
EQ-5D-5L VAS					
Baseline	50.5 (0.5)	50.5 (0.5)	0.03	0.97	-1.3 to 1.3
14-day	74.6 (0.5)	72.6 (0.5)	2.0	0.0048	0.6 to 3.3
28-day	79.9 (0.5)	78.8 (0.5)	1.1	0.092	-0.2 to 2.4
3-month	80.2 (0.5)	79.7 (0.5)	0.5	0.41	-0.7 to 1.8
6-month	80.4 (0.5)	79.2 (0.5)	1.2	0.075	-0.1 to 2.5

*Obtained from t-test

© 2026 Png ME et al. *JAMA Network Open*.

eTable 7. Incremental cost-effectiveness of nirmatrelvir-ritonavir versus usual care over six months from the sensitivity and subgroup analyses, in 2023/24 prices

	Nirmatrelvir-ritonavir: Usual care, n	Mean Costs (SE)		Incremental cost (bootstrap 95% CI)	Mean QALYs (SE)		Incremental QALYs (bootstrap 95% CI)	ICER, £/QALY	Probability of nirmatrelvir-ritonavir being cost-effective at specified cost-effectiveness threshold		
		Nirmatrelvir-ritonavir	Usual care		Nirmatrelvir-ritonavir	Usual care			£15 000	£20 000	£30 000
**Sensitivity analysis**											
Societal perspective	1736:1768	3938 (661.5)	3972 (381.1)	-34 (-1269 to 1201)	0.4144 (0.0023)	0.4026 (0.0018)	0.012 (0.0061 to 0.017)	-2 917 (SE quad)	0.65	0.68	0.74
Complete case analysis	1273:1213	1714 (753.4)	1282 (16.3)	432 (-58 to 922)	0.4244 (0.0018)	0.4167 (0.0015)	0.0077 (0.0022 to 0.013)	56 147 (NE quad)	0.10	0.14	0.21
**Subgroup analysis**											
Age with 65 years old as cutoff											
Less than 65 years old	1400:1418	3300 (832.1)	3343 (488.0)	-43 (-1479 to 1393)	0.4108 (0.0022)	0.3969 (0.0018)	0.014 (0.0078 to 0.02)	-3 108 (SE quad)	0.73	0.76	0.81
65 years old and above	336:350	3161 (450.6)	2261 (256.6)	900 (-1067 to 2866)	0.4299 (0.0025)	0.4288 (0.0022)	0.0011 (-0.0084 to 0.011)	818 017 (NE quad)	0.19	0.19	0.19
Age with 80 years old as cutoff											
Less than 80 years old	1720:1755	3284 (751.8)	3136 (437.9)	148 (-1186 to 1483)	0.4148 (0.0018)	0.4035 (0.0015)	0.011 (0.0061 to 0.016)	13 169 (NE quad)	0.59	0.63	0.70
80 years old and above	16:13	2197 (1128.5)	2192 (609.3)	5 (-3258 to 3268)	0.3974 (0.0149)	0.3684 (0.0127)	0.029 (-0.034 to 0.092)	169 (NE quad)	0.64	0.67	0.72
Comorbidity											
No	609:583	2809 (609.8)	1674 (317.0)	1136 (-311 to 2582)	0.4405 (0.0015)	0.4324 (0.0012)	0.0081 (0.001 to 0.015)	140 136 (NE quad)	0.058	0.068	0.09
Yes	1127:1185	3418 (841.8)	3845 (510.9)	-427 (-1903 to 1049)	0.4024 (0.0025)	0.3889 (0.0021)	0.014 (0.0066 to 0.02)	-31 568 (SE quad)	0.85	0.86	0.88
Lung disease											
No	1282:1329	3049 (677.5)	2476 (381.7)	572 (-712 to 1856)	0.4206 (0.0016)	0.4118 (0.0014)	0.0087 (0.0031 to 0.014)	65 706 (NE quad)	0.24	0.27	0.34
Yes	454:439	3789 (1023.0)	5104 (630.8)	-1315 (-3340 to 711)	0.3991 (0.0043)	0.3772 (0.0035)	0.022 (0.01 to 0.034)	-60 155 (SE quad)	0.93	0.94	0.95
Obesity											
No	1443:1456	3029 (713.3)	2951 (424.2)	78 (-1210 to 1366)	0.4215 (0.0017)	0.4103 (0.0015)	0.011 (0.0056 to 0.017)	7 029 (NE quad)	0.63	0.67	0.73
Yes	293:312	4188 (968.2)	3960 (524.0)	228 (-2012 to 2467)	0.3853 (0.0044)	0.3702 (0.0040)	0.015 (0.0014 to 0.029)	15 045 (NE quad)	0.52	0.56	0.61
Diabetes mellitus											
No	1604:1618	3237 (731.2)	3118 (427.3)	119 (-1205 to 1443)	0.4164 (0.0019)	0.4050 (0.0016)	0.011 (0.006 to 0.017)	10 377 (NE quad)	0.61	0.65	0.71
Yes	132:150	3189 (1074.1)	3249 (602.4)	-60 (-2156 to 2035)	0.3997 (0.0059)	0.3845 (0.0052)	0.015 (-0.0042 to 0.035)	-3 960 (SE quad)	0.66	0.69	0.72
Cardiovascular disease											

© 2026 Png ME et al. *JAMA Network Open*.

	Nirmatrelvir-ritonavir: Usual care, n	Mean Costs (SE)		Incremental cost (bootstrap 95% CI)	Mean QALYs (SE)		Incremental QALYs (bootstrap 95% CI)	ICER, £/QALY	Probability of nirmatrelvir-ritonavir being cost-effective at specified cost-effectiveness threshold		
		Nirmatrelvir-ritonavir	Usual care		Nirmatrelvir-ritonavir	Usual care			£15 000	£20 000	£30 000
No	1653:1705	3205 (745.4)	3124 (442.2)	81 (-1246 to 1409)	0.4153 (0.0018)	0.4036 (0.0015)	0.012 (0.0064 to 0.017)	6 945 (NE quad)	0.64	0.67	0.74
Yes	83:63	3779 (1120.4)	3269 (538.7)	511 (-2105 to 3126)	0.4158 (0.0080)	0.3940 (0.0066)	0.022 (-0.0039 to 0.048)	23 352 (NE quad)	0.49	0.52	0.58
Immune disorders											
No	1584:1602	3236 (748.3)	2993 (428.9)	243 (-1109 to 1596)	0.4167 (0.0018)	0.4056 (0.0015)	0.011 (0.0056 to 0.017)	21 978 (NE quad)	0.52	0.56	0.63
Yes	152:166	3256 (969.9)	4444 (615.3)	-1188 (-3537 to 1162)	0.3988 (0.0055)	0.3803 (0.0047)	0.019 (0.00058 to 0.036)	-64 073 (SE quad)	0.90	0.90	0.92
No. of dose of vaccination											
0	21:28	6812 (1575.4)	3796 (634.7)	3015 (-2280 to 8311)	0.3865 (0.0193)	0.3417 (0.0150)	0.045 (-0.019 to 0.11)	67 292 (NE quad)	0.18	0.21	0.27
1	16:7	6158 (2553.3)	9891 (1658.4)	-3733 (-10304 to 2838)	0.3007 (0.0430)	0.3166 (0.0410)	-0.016 (-0.1 to 0.071)	235 241 (SW quad)	0.87	0.85	0.83
2	78:84	4546 (1673.5)	5036 (1025.8)	-491 (-3660 to 2678)	0.4067 (0.0072)	0.3754 (0.0061)	0.031 (0.004 to 0.059)	-15 672 (SE quad)	0.74	0.77	0.82
3	656:693	2786 (739.5)	2896 (381.8)	-110 (-1547 to 1328)	0.4209 (0.0025)	0.4081 (0.0022)	0.013 (0.0047 to 0.021)	-8 607 (SE quad)	0.73	0.76	0.80
4	723:704	3642 (707.0)	2920 (439.5)	722 (-780 to 2224)	0.4125 (0.0026)	0.4049 (0.0023)	0.0076 (-0.0005 to 0.016)	95 515 (NE quad)	0.21	0.23	0.26
Major symptoms at baseline											
No	987:1045	2619 (661.6)	2704 (359.0)	-85 (-1349 to 1178)	0.4320 (0.0015)	0.4244 (0.0013)	0.0076 (0.0021 to 0.013)	-11 222 (SE quad)	0.69	0.71	0.75
Yes	749:723	4114 (911.1)	3742 (570.4)	372 (-1347 to 2091)	0.3892 (0.0032)	0.3726 (0.0026)	0.017 (0.0072 to 0.026)	22 419 (NE quad)	0.50	0.54	0.62
Duration of symptoms at baseline											
3 or less days	1262:1299	3222 (763.2)	3118 (446.1)	105 (-1308 to 1517)	0.4177 (0.0019)	0.4072 (0.0017)	0.011 (0.0048 to 0.016)	9 904 (NE quad)	0.60	0.63	0.69
More than 3 days	474:469	3333 (752.1)	3160 (434.0)	173 (-1359 to 1706)	0.4062 (0.0034)	0.3924 (0.0029)	0.014 (0.0035 to 0.024)	12 607 (NE quad)	0.55	0.59	0.66
Use of inhaled corticosteroid											
No	1268:1306	3069 (691.6)	2524 (379.4)	545 (-759 to 1850)	0.4188 (0.0017)	0.4122 (0.0015)	0.0066 (0.001 to 0.012)	82 071 (NE quad)	0.24	0.26	0.31
Yes	468:462	3797 (990.9)	4838 (636.3)	-1042 (-3018 to 934)	0.4022 (0.0038)	0.3779 (0.0032)	0.024 (0.013 to 0.035)	-42 942 (SE quad)	0.91	0.92	0.94
Vaccination status											
Not vaccinated	21:28	6812 (1575.4)	3796 (634.7)	3015 (-2280 to 8311)	0.3865 (0.0193)	0.3417 (0.0150)	0.045 (-0.019 to 0.11)	67 292 (NE quad)	0.18	0.21	0.27

© 2026 Png ME et al. *JAMA Network Open*.

	Nirmatrelvir-ritonavir: Usual care, n	Mean Costs (SE)		Incremental cost (bootstrap 95% CI)	Mean QALYs (SE)		Incremental QALYs (bootstrap 95% CI)	ICER, £/QALY	Probability of nirmatrelvir-ritonavir being cost-effective at specified cost-effectiveness threshold		
		Nirmatrelvir-ritonavir	Usual care		Nirmatrelvir-ritonavir	Usual care			£15 000	£20 000	£30 000
Vaccinated	1715:1740	3205 (752.5)	3118 (437.8)	87 (-1246 to 1421)	0.4152 (0.0019)	0.4042 (0.0015)	0.011 (0.0057 to 0.016)	7 957 (NE quad)	0.63	0.67	0.73
Time elapsed since most recent dose											
Not vaccinated	21:28	6812 (1575.4)	3796 (634.7)	3015 (-2280 to 8311)	0.3865 (0.0193)	0.3417 (0.0150)	0.045 (-0.019 to 0.11)	67 292 (NE quad)	0.18	0.21	0.27
0 to 3 months	320:295	2620 (587.3)	2221 (327.7)	400 (-761 to 1560)	0.4106 (0.0029)	0.4101 (0.0027)	0.00046 (-0.012 to 0.012)	871 442 (NE quad)	0.26	0.27	0.28
3 to 6 months	353:366	2926 (710.5)	3234 (442.9)	-308 (-1856 to 1240)	0.4209 (0.0029)	0.4079 (0.0027)	0.013 (0.0031 to 0.023)	-23 718 (SE quad)	0.76	0.78	0.82
More than 6 months	978:1004	3442 (806.5)	3273 (448.6)	169 (-1426 to 1764)	0.4156 (0.0025)	0.4023 (0.0020)	0.013 (0.0063 to 0.02)	12 693 (NE quad)	0.57	0.61	0.68
Swab positivity status											
PCR positive	584:629	3462 (953.5)	3417 (565.8)	45 (-1633 to 1722)	0.4071 (0.0030)	0.3983 (0.0025)	0.0088 (-0.00047 to 0.018)	5 064 (NE quad)	0.60	0.62	0.66
LFD positive	1152:1139	3147 (656.9)	2969 (377.5)	177 (-1144 to 1499)	0.4186 (0.0019)	0.4060 (0.0017)	0.013 (0.0065 to 0.019)	14 105 (NE quad)	0.57	0.61	0.68
Risk category											
2	16:13	2339 (1084.7)	2192 (623.3)	147 (-2914 to 3208)	0.3939 (0.0149)	0.3684 (0.0130)	0.025 (-0.041 to 0.092)	5 779 (NE quad)	0.56	0.59	0.63
3	51:42	2457 (898.2)	2735 (598.3)	-279 (-2705 to 2147)	0.4255 (0.0073)	0.4233 (0.0066)	0.0022 (-0.029 to 0.033)	-126 839 (SE quad)	0.59	0.59	0.59
4	113:121	5468 (692.6)	1588 (273.1)	3879 (-1457 to 9215)	0.4258 (0.0041)	0.4352 (0.0034)	-0.0094 (-0.026 to 0.0072)	-414 324 (NW quad)	0.013	0.013	0.012
5	156:174	1816 (363.9)	2620 (235.2)	-804 (-2455 to 848)	0.4374 (0.0032)	0.4301 (0.0030)	0.0073 (-0.0056 to 0.02)	-109 955 (SE quad)	0.86	0.87	0.88
6	940:978	3619 (930.8)	4097 (557.9)	-478 (-2127 to 1171)	0.3984 (0.0029)	0.3816 (0.0023)	0.017 (0.0089 to 0.025)	-28 590 (SE quad)	0.86	0.87	0.89
7	125:100	2250 (923.5)	1669 (518.0)	581 (-1300 to 2463)	0.4424 (0.0045)	0.4365 (0.0036)	0.0059 (-0.0091 to 0.021)	98 118 (NE quad)	0.28	0.29	0.33
8	165:179	2237 (508.1)	1732 (253.8)	505 (-695 to 1705)	0.4427 (0.0027)	0.4311 (0.0022)	0.012 (-0.001 to 0.024)	43 413 (NE quad)	0.28	0.29	0.41
9	170:161	2702 (741.1)	1590 (432.4)	1112 (-344 to 2568)	0.4348 (0.0032)	0.4274 (0.0027)	0.0074 (-0.0068 to 0.022)	150 763 (NE quad)	0.062	0.074	0.10

ICER: incremental cost-effectiveness ratio, LFD: lateral flow device, NE: north-east, NW: north-west, PCR: polymerase chain reaction, QALY: quality-adjusted life year, quad: quadrant of the cost-effectiveness plane, SE: south-east. NHS risk category 2: aged ≥80 years; NHS risk category 3: aged 75-79 years; NHS risk category 4: aged 70-74 years; NHS risk category 5: aged 65-69 years; NHS risk category 6: aged 18-64 years in an at-risk group; NHS risk category 7: Aged 60-64 years and not in an at-risk group; NHS risk category 8: Aged 55-59 years and not in an at-risk group; NHS risk category 9: Aged 50-54 years and not in an at-risk group. All base case, sensitivity and subgroup analyses were adjusted using age, vaccination status and comorbidity except the following.

© 2026 Png ME et al. *JAMA Network Open*.

Those aged 80 and above were adjusted by comorbidity. Other age groups were adjusted by vaccination status and comorbidity. NHS priority category was adjusted by age and vaccination status. Number of doses of vaccination was adjusted by age and comorbidity. Those with diabetes, cardiovascular disease, immune disorder, had one dose of vaccination, or were in NHS risk category 8 or 9, were adjusted by age.

© 2026 Png ME et al. *JAMA Network Open*.

eTable 8. Summary of the cost-effectiveness of nirmatrelvir-ritonavir by age group and risk status under the £20 000 per QALY threshold

Age group (years)	At-risk	Not at-risk
18-49	CE	Not eligible
50-54	CE	Not CE
55-59	CE	Not CE
60-64	CE	Not CE
65-69	CE	CE
70-74	Not CE	Not CE
75-79	CE	CE
80+	CE	CE

CE: cost-effective, “Not eligible” indicates age-risk groups that did not meet the PANORAMIC trial eligibility criteria and were therefore not included in the analysis; cost-effectiveness was not evaluated for these groups.

© 2026 Png ME et al. *JAMA Network Open*.

eTable 9. Summary of the cost-effectiveness of nirmatrelvir-ritonavir by vaccination status under the £20 000 per QALY threshold

Vaccination group	CE status
Unvaccinated	Not CE
1 dose	CE
2 doses	CE
3 doses	CE
4 doses	Not CE
0-3 months since most recent dose	Not CE
3-6 months since most recent dose	CE
>6 months since most recent dose	CE

CE: cost-effective

© 2026 Png ME et al. *JAMA Network Open*.

eReferences

1 BNF. Nirmatrelvir with ritonavir: Medicinal forms. 2025. https://bnf.nice.org.uk/drugs/nirmatrelvir-with-ritonavir/medicinal-forms/ (accessed August 28, 2025).

2 National Health Service. National Cost Collection for the NHS. 2024. https://www.england.nhs.uk/costing-in-the-nhs/national-cost-collection/ (accessed June 20, 2025).

3 Multicentre Aneurysm Screening Study Group. Multicentre aneurysm screening study (MASS): cost effectiveness analysis of screening for abdominal aortic aneurysms based on four year results from randomised controlled trial. *BMJ* 2002; 325: 1135.

4 NHS Employers. Pay scales for 2023/24 [Archived]. 2023. https://www.nhsemployers.org/articles/pay-scales-202324-archived (accessed April 24, 2025).

5 Jones K, Weatherly H, Birch S, *et al.* Unit Costs of Health and Social Care 2024 Manual. 2025.

6 Bluecrest. Full body private health checks. 2024. ttps://www.bluecrestwellness.com/packages (accessed June 19, 2025).

7 National Health Service. Chiropractic. 2024. https://www.nhs.uk/conditions/chiropractic (accessed Oct 30, 2024).

8 Lymphoma Association. Complementary therapy. 2015. https://www.nhs.uk/ipgmedia/National/Lymphoma%20Association/assets/Complementarytherapy.pdf (accessed Oct 23, 2022).

9 Local Government Association. LGA pay and grading structure 2024. 2025. https://www.local.gov.uk/about/who-we-are-and-what-we-do/what-we-spend-and-how-we-spend-it/organisational-information/lga (accessed June 19, 2025).

10 British Council. UK Pay Policy. 2022. https://www.britishcouncil.org/about-us/how-we-work/policies/uk-pay-policy (accessed Oct 23, 2022).

11 Curtis L, Burns A. Unit Costs of Health and Social Care 2015. 2015. DOI:https://doi.org/10.22024/UniKent/01.02.70995.

12 National Health Service. NHS prescription charges. 2021. https://www.nhs.uk/nhs-services/prescriptions-and-pharmacies/nhs-prescription-charges/ (accessed April 20, 2023).

13 Prospects. Herbalist. 2025. https://www.prospects.ac.uk/job-profiles/herbalist (accessed June 19, 2025).

14 National Health Service. Archived Reference Costs. 2020. https://improvement.nhs.uk/resources/reference-costs/ (accessed March 24, 2020).

15 Curtis L. Unit Costs of Health and Social Care 2013. 2013. https://www.pssru.ac.uk/project-pages/unit-costs/unit-costs-2013 (accessed Oct 23, 2022).

16 Glendinning C, Jones K, Baxter K, *et al.* Home Care Re-ablement Services: Investigating the longer-term impacts (prospective longitudinal study). 2010.

17 Murphy J, Halloran S, Gray A. Cost-effectiveness of the faecal immunochemical test at a range of positivity thresholds compared with the guaiac faecal occult blood test in the NHS Bowel Cancer Screening Programme in England. *BMJ Open* 2017; 7: e017186.

© 2026 Png ME et al. *JAMA Network Open*.

18 Health Innovation Network. Rapid Evaluation of Croydon Virtual Ward. 2021. https://healthinnovationnetwork.com/wp-content/uploads/2022/01/Croydon-VW-Evaluation-Report-to-NHSX-v10.pdf (accessed Oct 23, 2022).

19 Curtis L, Burns A. Unit Costs of Health and Social Care 2020. 2020. https://www.pssru.ac.uk/project-pages/unit-costs/unit-costs-2020/ (accessed March 18, 2022).

20 Office for National Statistics. Employee earnings in the UK: 2024. 2024. https://www.ons.gov.uk/employmentandlabourmarket/peopleinwork/earningsandworkinghours/bulletins/annualsurveyofhoursandearnings/2024 (accessed May 14, 2025).

21 Office for National Statistics. Gender pay gap in the UK: 2024. 2024. https://www.ons.gov.uk/employmentandlabourmarket/peopleinwork/earningsandworkinghours/bulletins/genderpaygapintheuk/2024 (accessed May 14, 2025).

© 2026 Png ME et al. *JAMA Network Open*.
