# Supplementary material for: Economic Evaluation of Oral Nirmatrelvir-Ritonavir for COVID-19 in Higher Risk Outpatients
Source: JAMA Netw Open. 2026 May 6;9(5):e2612381. doi: 10.1001/jamanetworkopen.2026.12381 (PMC13150634; doi:10.1001/jamanetworkopen.2026.12381)
Supplement: Supplement 2. — Nonauthor Collaborators [file jamanetwopen-e2612381-s002.pdf]

Supplemental Online Content: Nonauthor Collaborators
*First name, last name, and suffix (if applicable) are required and will appear in PubMed.

***Group Name(s): PANORAMIC Trial Collaborative Group**

***First Name and Middle Initial(s)**	***Last Name**	***Suffix (eg, Jr, III)**	Academic Degrees	Institution	Location (city, state/province, country)	Role or Contribution, eg, chair, principal investigator	Group (if more than 1 Group listed in the byline) and/or Subgroup (eg, Steering Committee)
Oghenekome A	Gbinigie		DPhil	Nuffield Department of Primary Care Health Sciences, University of Oxford	Oxford, UK	TMG member	PANORAMIC Trial Collaborative Group
Najib M	Rahman		DPhil	Oxford Respiratory Trials Unit, University of Oxford	Oxford, UK	TMG member	PANORAMIC Trial Collaborative Group
Gail	Hayward		DPhil	Nuffield Department of Primary Care Health Sciences, University of Oxford	Oxford, UK	TMG member	PANORAMIC Trial Collaborative Group
Duncan B	Richards		DM	Nuffield Department of Orthopaedics, Rheumatology and Musculoskeletal Sciences, University of Oxford	Oxford, UK	TMG member	PANORAMIC Trial Collaborative Group
Jienchi	Dorward		MBChB	Nuffield Department of Primary Care Health Sciences, University of Oxford	Oxford, UK	TMG member	PANORAMIC Trial Collaborative Group
David M	Lowe		PhD	Institute of Immunity and Transplantation, University College London	London, UK	TMG member	PANORAMIC Trial Collaborative Group
Joseph F	Standing		PhD	UCL Great Ormond Street Institute of Child Health	London, UK	TMG member	PANORAMIC Trial Collaborative Group
Judith	Breuer		MD	UCL Great Ormond Street Institute of Child Health	London, UK	TMG member	PANORAMIC Trial Collaborative Group
Saye	Khoo		FRCP	Department of Pharmacology, University of Liverpool	Liverpool, UK	TMG member	PANORAMIC Trial Collaborative Group
Kerenza	Hood		PhD	Centre for Trials Research, Cardiff University	Cardiff, UK	TMG member	PANORAMIC Trial Collaborative Group
Jonathan S	Nguyen-Van-Tam		FMedSci	University of Nottingham School of Medicine	Nottingham, UK	TMG member	PANORAMIC Trial Collaborative Group
Mahendra G	Patel		PhD	Nuffield Department of Primary Care Health Sciences, University of Oxford	Oxford, UK	TMG member	PANORAMIC Trial Collaborative Group
Benjamin R	Saville		PhD	Berry Consultants	Austin, TX, USA	TMG member	PANORAMIC Trial Collaborative Group
Joe	Marion		PhD	Berry Consultants	Austin, TX, USA	TMG member	PANORAMIC Trial Collaborative Group
Nick	Francis		PhD	Primary Care Research Centre, University of Southampton	Southampton, UK	TMG member	PANORAMIC Trial Collaborative Group
Nicholas P B	Thomas		FRCGP	Windrush Medical Practice	Witney, UK	TMG member	PANORAMIC Trial Collaborative Group
Philip	Evans		FRCGP	University of Exeter	Exeter, UK	TMG member	PANORAMIC Trial Collaborative Group
Melissa	Dobson		BSc	Oxford Respiratory Trials Unit, University of Oxford	Oxford, UK	TMG member	PANORAMIC Trial Collaborative Group
Jane	Holmes		PhD	Nuffield Department of Primary Care Health Sciences, University of Oxford	Oxford, UK	TMG member	PANORAMIC Trial Collaborative Group
Victoria	Harris		PhD	Nuffield Department of Orthopaedics, Rheumatology and Musculoskeletal Sciences, University of Oxford	Oxford, UK	TMG member	PANORAMIC Trial Collaborative Group
Mark	Lown		PhD	Primary Care Research Centre, University of Southampton	Southampton, UK	TMG member	PANORAMIC Trial Collaborative Group
Oliver	van Hecke		DPhil	Nuffield Department of Primary Care Health Sciences, University of Oxford	Oxford, UK	TMG member	PANORAMIC Trial Collaborative Group
Michelle A	Detry		PhD	Berry Consultants	Austin, TX, USA	TMG member	PANORAMIC Trial Collaborative Group
Christina T	Saunders		PhD	Berry Consultants	Austin, TX, USA	TMG member	PANORAMIC Trial Collaborative Group
Mark	Fitzgerald		PhD	Berry Consultants	Austin, TX, USA	TMG member	PANORAMIC Trial Collaborative Group
Nicholas S	Berry		PhD	Berry Consultants	Austin, TX, USA	TMG member	PANORAMIC Trial Collaborative Group
Sam	Mort		PGCert	Nuffield Department of Primary Care Health Sciences, University of Oxford	Oxford, UK	TMG member	PANORAMIC Trial Collaborative Group
Bhautesh D	Jani		PhD	University of Glasgow	Glasgow, UK	TMG member	PANORAMIC Trial Collaborative Group
Nigel D	Hart		MD	Queen's University Belfast	Belfast, UK	TMG member	PANORAMIC Trial Collaborative Group
Haroon	Ahmed		PhD	Cardiff University	Cardiff, UK	TMG member	PANORAMIC Trial Collaborative Group
Daniel	Butler		MBChB	Queen's University Belfast	Belfast, UK	TMG member	PANORAMIC Trial Collaborative Group
Micheal	McKenna		BSc	Nuffield Department of Primary Care Health Sciences, University of Oxford	Oxford, UK	TMG member	PANORAMIC Trial Collaborative Group
Lucy	Cureton		BSc	Nuffield Department of Primary Care Health Sciences, University of Oxford	Oxford, UK	TMG member	PANORAMIC Trial Collaborative Group
Meena	Patil			Nuffield Department of Primary Care Health Sciences, University of Oxford	Oxford, UK	TMG member	PANORAMIC Trial Collaborative Group
Monique	Andersson		MD	Oxford University Hospitals NHS Foundation Trust	Oxford, UK	TMG member	PANORAMIC Trial Collaborative Group
Clare	Bateman		BA	Nuffield Department of Primary Care Health Sciences, University of Oxford	Oxford, UK	TMG member	PANORAMIC Trial Collaborative Group
Jennifer C	Davies		PhD	Nuffield Department of Primary Care Health Sciences, University of Oxford	Oxford, UK	TMG member	PANORAMIC Trial Collaborative Group
Andrew	Ustianowski		PhD	North Manchester General Hospital	Manchester, UK	TMG member	PANORAMIC Trial Collaborative Group
Andrew Carson	Stevens		PhD	Cardiff University	Cardiff, UK	TMG member	PANORAMIC Trial Collaborative Group
