# Supplementary material for: Economic Evaluation of Oral Nirmatrelvir-Ritonavir for COVID-19 in Higher Risk Outpatients
Source: JAMA Netw Open. 2026 May 6;9(5):e2612381. doi: 10.1001/jamanetworkopen.2026.12381 (PMC13150634; doi:10.1001/jamanetworkopen.2026.12381)
Supplement: Supplement 3. — Data Sharing Statement [file jamanetwopen-e2612381-s003.pdf]

Data Sharing Statement

Png. Economic Evaluation of Oral Nirmatrelvir-Ritonavir for COVID-19 in Higher Risk Outpatients. *JAMA Netw Open*. Published May 06, 2026.
doi:10.1001/jamanetworkopen.2026.12381

Data
Data available: No

Additional Information
Explanation for why data not available: Qualifying researchers who wish to access our data should submit a proposal with a valuable research question. Proposals will be assessed by a committee formed from the trial management group, including senior statistical and clinical representation. Data will be shared in accordance with the data sharing policy of Nuffield Department of Primary Care Health Sciences.
